# Characterization of *vegetative inflorescence* (*mc-vin*) mutant provides new insight into the role of *MACROCALYX* in regulating inflorescence development of tomato

**DOI:** 10.1038/srep18796

**Published:** 2016-01-04

**Authors:** Fernando J. Yuste-Lisbona, Muriel Quinet, Antonia Fernández-Lozano, Benito Pineda, Vicente Moreno, Trinidad Angosto, Rafael Lozano

**Affiliations:** 1 Centro de Investigación en Biotecnología Agroalimentaria (BITAL), Universidad de Almería, 04120 Almería, Spain; 2 Instituto de Biología Molecular y Celular de Plantas (UPV-CSIC), Universidad Politécnica de Valencia. Avenida de los Naranjos s/n. 46022 Valencia, Spain

## Abstract

Inflorescence development is a key factor of plant productivity, as it determines flower number. Therefore, understanding the mechanisms that regulate inflorescence architecture is critical for reproductive success and crop yield. In this study, a new mutant, *vegetative inflorescence* (*mc-vin*), was isolated from the screening of a tomato (*Solanum lycopersicum* L.) T-DNA mutant collection. The *mc-vin* mutant developed inflorescences that reverted to vegetative growth after forming two to three flowers, indicating that the mutated gene is essential for the maintenance of inflorescence meristem identity. The T-DNA was inserted into the promoter region of the *MACROCALYX* (*MC*) gene; this result together with complementation test and expression analyses proved that *mc-vin* is a new knock-out allele of *MC*. Double combinations between *mc-vin* and *jointless* (*j*) and *single flower truss* (*sft*) inflorescence mutants showed that *MC* has pleiotropic effects on the reproductive phase, and that it interacts with *SFT* and *J* to control floral transition and inflorescence fate in tomato. In addition, *MC* expression was mis-regulated in *j* and *sft* mutants whereas *J* and *SFT* were significantly up-regulated in the *mc-vin* mutant. Together, these results provide new evidences about *MC* function as part of the genetic network regulating the development of tomato inflorescence meristem.

The transition from vegetative growth to reproductive development is a crucial event of morphogenesis in flowering plants, which involves a remarkable change in the developmental program of the shoot apical meristem (SAM). This meristem first produces leaves, buds, and stem; but once SAM acquires a reproductive competence, it forms an inflorescence meristem (IM), which in turn produces floral meristems (FM) that give rise to flowers. The knowledge on the genetic basis of floral transition derives mainly from studies in monopodial species such as *Arabidopsis thaliana* and *Antirrhinum majus*, where floral transition occurs once in a plant’s life and it is at this point that the indeterminate SAM generates vegetative or reproductive organs on its flanks (reviewed in^1^,^2^,^3^,^4^). In contrast, the genetic control of the transition to flowering in sympodial species is still poorly understood. Tomato (*Solanum lycopersicum* L.) is an autonomously flowering plant, which as well as being an important commercial crop, has become a model species for the study of flowering process of plants with a sympodial growth pattern (reviewed in^5^,^6^,^7^,^8^). The primary shoot (or initial segment) of tomato plants is determinate and the SAM is completely consumed in the development of the first inflorescence. At floral transition, SAM terminates in IM and produces a new IM on its side before differentiating into a FM. Reiteration and rapid termination of IMs, which are developed perpendicular to one another, produce an inflorescence organized in a zigzag pattern until the production of about five to ten flowers. At the same time, vegetative growth continues from the axil of the youngest leaf through a sympodial vegetative meristem (SM), whose development is boosted and displaces the inflorescence laterally. SM generally produces three leaves before terminating with a new inflorescence. This growth pattern is repeated by the formation of successive determinate units or sympodial segments; thus, the architecture of a tomato plant means a regular alternation of vegetative and reproductive phases resulting from the newly arisen SM (reviewed in^7^).

Although knowledge about genetic control of flowering in tomato remains fragmentary compared to other model plants, the characterization of several mutants has allowed for a deeper understanding of this process in this species (reviewed in^5^,^6^,^7^,^9^,^10^,^11^). Many spontaneous mutants have been preserved and characterized by the Tomato Genetic Resource Center^12^. What is more, new mutant populations generated by chemical and physical mutagens have been used to identify novel candidate genes involved in tomato reproduction (exhaustive data can be found on these Websites: http://tgrc.ucdavis.edu/ and http://zamir.sgn.cornell.edu/mutants/). Among others, important genes for inflorescence architecture have been found through the study of different mutants. Thus, the *falsiflora* (*fa)* mutant produces compound inflorescences made of leafy shoots suggesting that *FA* is an FM identity gene like its *Arabidopsis* ortholog *LEAFY*^13^. The *anantha* (*an*) mutant produces inflorescences reminiscent of the common cauliflower^14^ and the *compound inflorescence* (*s*) mutant inflorescences are highly branched, but eventually bear up to 200 fertile flowers^9^,^15^. Both *AN* and *S* were suggested to control inflorescence architecture by promoting successive stages in the progression of an IM to FM^9^. *S* encodes a homeobox transcription factor and *AN* is orthologous to the *Arabidopsis UNUSUAL FLORAL ORGANS* gene^9^. On the other hand, the *TERMINATING FLOWER* (*TMF*) gene, which belongs to the ALOG gene family, was suggested to synchronize the flowering transition. Thereby, in contrast to *s*, the *tmf* mutant produces a single-flower primary inflorescence^16^. This phenotype is due to the precocious activation of a conserved floral specification complex encoded by *AN* and *FA* in the *tmf* mutant.

In addition, the inflorescence architecture is also disrupted in the *jointless* (*j*) and *single flower truss* (*sft*) mutants. The *j* mutant was first characterized by the lack of pedicel abscission zone although it also produces indeterminate inflorescences that revert to a vegetative growth after production of two or three flowers^15^,^17^,^18^. The *J* gene encodes a MADS-box transcription factor and belongs to the same clade as the *Arabidopsis SHORT VEGETATIVE PHASE* (*SVP*) and *AGAMOUS*-*LIKE 24* (*AGL24*) genes^19^. In addition to its late flowering phenotype, the *sft* mutant also shows reversion of the inflorescence towards vegetative functioning after the initiation of one or few flowers depending on the growing conditions^15^,^20^,^21^. The growing of the *sft* inflorescence may disrupt normal tomato sympodial growth^20^,^21^. *SFT* is the orthologue of the *Arabidopsis FLOWERING LOCUS T* (*FT*) gene and encodes a strong flowering promoter, which is graft-transmissible^20^,^21^,^22^. Double mutant analyses showed that *J* and *SFT* cooperatively regulate the architecture of the inflorescence to prevent early change of IM identity once inflorescence morphogenesis is initiated^11^. The *macrocalyx* (*mc*) mutant also displays reverted inflorescences with only few flowers with leafy-like sepals^23^. The *MC* gene belongs to the *APETALA1/FRUITFULL* (*AP1/FUL*) subfamily of the MADS-box gene family and is closely linked to the *RIPENING INHIBITOR* (*RIN*) gene that regulates fruit ripening^23^. In addition to its role in inflorescence and sepal development, MC controls pedicel abscission zone development by forming heterodimer with J^24^,^25^,^26^.

Despite the identification of these genes, the main disadvantage for both spontaneous and induced mutants is the difficulty to isolate the mutated gene, which requires positional cloning and/or genome sequencing strategies. Insertional mutagenesis comes to help solve this problem, because mutated gene is tagged by transposon or T-DNA insertions. As the sequence of the inserted element is known, the genomic sequences flanking the insertion can easily be identified using various cloning and PCR-based strategies. However, few insertional tomato mutants have been described to date. A collection of tomato enhancer trapping insertional mutants has been generated by using the binary vector pD991^27^. This report showed the molecular and functional characterization of a new tomato T-DNA mutant, *vegetative inflorescence* (*mc-vin*), which displayed a reversion of the IM to vegetative growth after producing a few flowers with homeotic conversion from sepals to leaf-like structures. The cloning of the tagged gene has revealed that the T-DNA was inserted into the promoter region of the *MC* gene, which was firstly described as involved in sepal development^23^. Additionally, the role of *MC* gene in pedicel abscission zone has been extensively investigated^24^,^25^,^26^; however, its function in inflorescence meristem remains to be elucidated. With the aim to better understand the genetic basis of tomato inflorescence development, the interactions among *MC*, *J*, and *SFT* genes have been analysed by using *in situ* hybridization and double mutant analysis. Results indicate that *MC* is a crucial component of the genetic pathway regulating inflorescence development of tomato, and that this function is exerted through its interaction with *SFT* and *J* genes.

## Results

### Isolation and phenotypic characterization of *mc-vin* mutant

**Figure 1:**
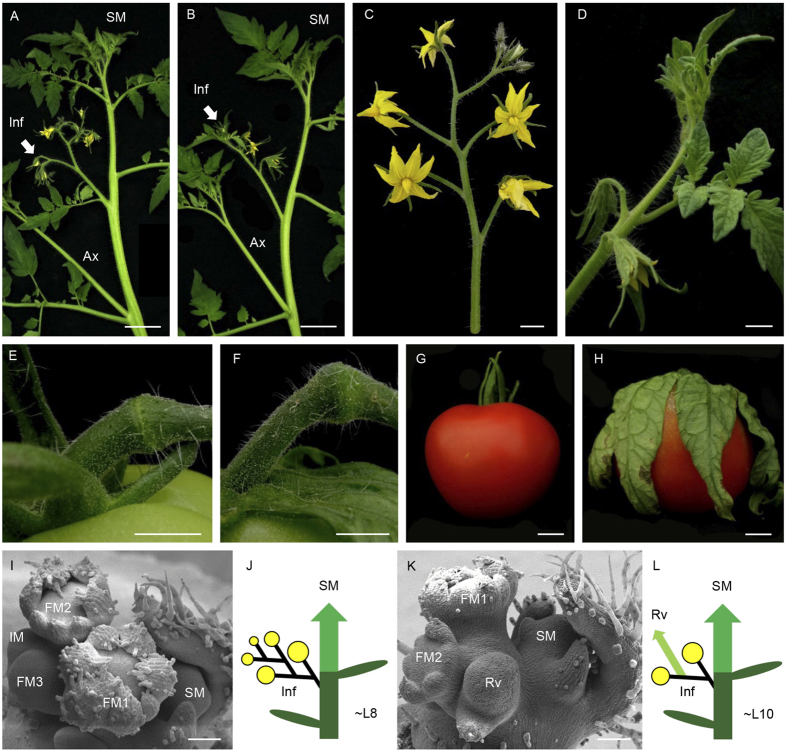
Comparison of wild type (WT) Moneymaker cultivar and *vegetative inflorescence* (*mc-vin*) mutant phenotypes of tomato. (**A**,**B**) apical shoot of (**A**) WT plant and (**B**) *mc-vin* mutant showing one inflorescence (Inf, arrow) and the sympodial growth development (SM). An axillary shoot (Ax) develops at the axil of the leaf under inflorescence. (**C**) WT inflorescence containing 9 flowers. (**D**) *mc-vin* mutant inflorescence that reverted to a vegetative growth after 2 flowers with leaf-like sepals. (**E**) complete WT pedicel abscission zone. (**F**) Incomplete *mc-vin* mutant pedicel abscission zone. (**G**) WT tomato mature fruit with sepals remaining attached to the fruit. (**H**) *mc-vin* mutant mature fruit with leaf-like sepals remaining attached to the fruit. (**I**) Scanning electron microscopy (SEM) image of a WT inflorescence containing three flower buds (FM1 to FM3) and an inflorescence meristem (IM). Note the development of the sympodial meristem (SM) at the axil of the last formed leaf. (**J**) Diagram of a WT plant (yellow closed circles: flowers, central column: main shoot composed of different sympodial segments, green ovals: leaves). The initial segment is composed of around 8 leaves and the first inflorescence (Inf). This inflorescence is displaced laterally by the outgrowth of the first sympodial segment (SM) at the axil of the last formed leaf. (**K**) SEM image of a *mc-vin* mutant inflorescence containing two flower buds (FM1, FM2) before initiating a reverted vegetative meristem (Rv). Note the development of the sympodial meristem (SM) at the axil of the last formed leaf. (**L**) Diagram of a *mc-vin* mutant plant (symbols and annotations are the same as indicated in J, small light green arrow: reverted vegetative meristem (Rv) inside the inflorescence). Scale bars: 5 cm in A and B; 1 cm in C-H; and 100 μm in I and K.

As part of a systematic analysis of gene function involved in tomato reproductive development, the *vegetative inflorescence* (*mc-vin*) mutant was isolated from the screening of a collection of T2 segregating T-DNA lines generated from the tomato cultivar Moneymaker (MM). The *mc-vin* mutant exhibited abnormal inflorescence development compared to wild type (WT) (Fig. 1) but the mutation did not affect the tomato sympodial growth (Fig. 1A,B). Tomato WT inflorescences usually contained about 5-10 open fertile flowers (Fig. 1C) while *mc-vin* produced indeterminate inflorescences that reverted to a vegetative growth after the formation of some fertile flowers showing long leafy-like sepals (Fig. 1D). These leafy sepals remained attached to the fruit during its development and maturation (Fig. 1H). The mutation also affected the pedicel abscission zone development since although present, the abscission zone in floral and fruit pedicel of *mc-vin* mutant was incomplete compared to WT ones (Fig. 1E,F).

Microscopic observations were performed so as to compare the early morphogenesis of WT and mutant inflorescences in depth. At floral transition, after initiation of about 8 leaves, the SAM of WT tomato initiated the inflorescence by producing the first FM, and a lateral IM was formed adjacently. While FM matured into a flower, IM produced one new lateral IM before transitioning to the next FM and so on. Each new IM developed perpendicularly to the one formed previously resulting in the familiar zigzag pattern of tomato inflorescences (Fig. 1I). At the same time, SM developed at the axil of the last formed leaf (Fig. 1I, J). In the *mc-vin* mutant, although floral transition took place somewhat later, inflorescence initiation occurred as in WT but after the production of about two flowers, a reverted vegetative meristem was visible in the normal location of the IM (Fig. 1K). Sympodial development was not affected by the *mc-vin* mutation (Fig. 1K, L).

Genetic analysis performed on T2 progeny - of which 13 plants were WT and 3 plants displayed mutant phenotype - showed that the segregation observed was consistent with a monogenic recessive inheritance for the *mc-vin* mutant phenotype (χ^2^ = 0.33, *P* = 0.56).

### Molecular analysis of the *mc-vin* mutant

**Figure 2:**
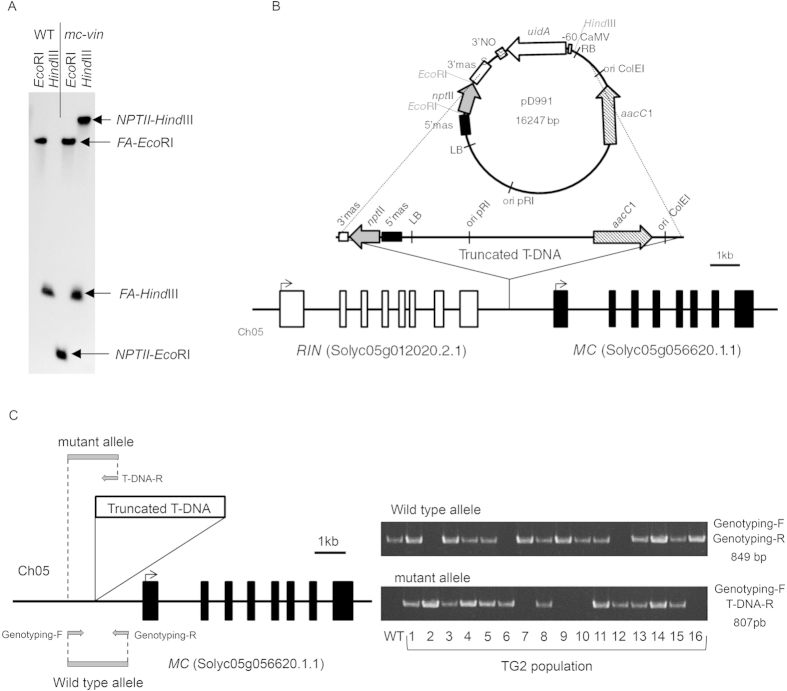
Characterization of the T-DNA insertion site in the *vegetative inflorescence* (*mc-vin*) mutant. (**A**) Southern blot analysis using a chimeric probe fusing the complete coding sequence of the *NEOMYCIN PHOSPHOTRANSFERASE II (NPTII)* gene to 811 pb of coding sequence of *FALSIFLORA (FA)* gene (used as hybridization positive control). (**B**) Genomic organization of the *RIPENING INHIBITOR (RIN)* and *MACROCALYX (MC)* genes and the T-DNA insertion in the *mc-vin* mutant. *RIN* and *MC* exons are depicted as white and black boxes, respectively. The truncated T-DNA insertion contains the left border (LB) and two genes (*nptII*, coding for neomycin phosphotransferase II; *aacC1* coding for acetyl-CoA carboxylase). (**C**) Primer set used for genotyping the T2 population. The specific genomic forward (Genotyping-F) and reverse (Genotyping-R) primers to amplify WT allele (without T-DNA insertion). The specific genomic forward (Genotyping-F) and the specific T-DNA border primer (T-DNA-R) to amplify the mutant allele (carrying the T-DNA insertion). T2 plants heterozygous (1, 3, 4, 5, 8, 11, 13, 14, and 15) and homozygous for the WT allele (7, 9, 10, and 16) showed WT phenotype, while T2 plants homozygous for the mutant allele (2, 6, and 12) displayed *mc-vin* mutant phenotype.

In order to determine the gene affected by the *mc-vin* mutation, the T-DNA insertion site was identified. Southern blot analysis showed that the original T1 plant carried a single T-DNA insertion (Fig. 2A), which was consistent with the results from the genetics analysis. Anchor-PCR protocol^28^,^29^ was used to locate the insertion site of T-DNA into the tomato genome. The amplification and cloning of genomic regions flanking the T-DNA insertion showed that it was located on chromosome 05, between *RIN* (Solyc05g012020.2.1) and *MC* (Solyc05g056620.1.1) genes. The T-DNA was inserted 1,265 bp upstream of the translation start codon of the *MC* gene and 1,198 bp downstream of the stop codon of the *RIN* gene (Fig. 2B). During the insertional process, the T-DNA underwent some rearrangements since the right border and the *uidA* reporter gene were removed. In contrast, 11,055 bp of the pD991 vector backbone were added to the left border. Thus, a truncated T-DNA fragment encompassing a total of 13,434 bp was inserted into the *mc-vin* mutant (Fig. 2B). Co-segregation analysis showed that all mutant plants carried the T-DNA insertion in the homozygous state (Fig. 2C), indicating that the mutant phenotype was associated with the insertion.

**Figure 3:**
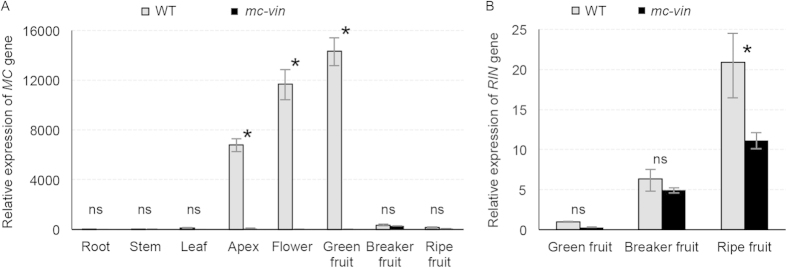
Expression of *MACROCALYX (MC)* and *RIPENING INHIBITOR (RIN)* in the wild type (WT) Moneymaker cultivar and the *vegetative inflorescence* (*mc-vin*) mutant. (A) Relative qRT-PCR expression analysis of the *MC* gene in different plant tissues. (**B**) Relative qRT-PCR expression analysis of the *RIN* gene along fruit development. ns, no statistically significant differences; *significant differences at *P *< 0.05.

qRT-PCR was used to analyse the spatial expression pattern of *MC* and *RIN* genes in WT and *mc-vin*. While *MC* transcripts were accumulated in apex, flower, and green fruit tissues of WT plants, no expression of *MC* gene was detected in any vegetative or reproductive tissue of mutant plants (Fig. 3A). The complete abolishment of *MC* expression in *mc-vin* plants cannot be attributable to putative lesions in the transcribed region promoted by the T-DNA insertion as no differences in the sequence of the *MC* genomic region were detected in the *mc-vin* mutant respect to WT. Regarding the *RIN* gene, although there were differences of expression levels depending on the fruit developmental stage, it was expressed in fruits of both WT and *mc-vin* (Fig. 3B). Hence, the results indicated that the tagged gene is most likely *MC* and that the *mc-vin* mutation is either in the promoter of *MC* or in another domain regulating *MC* expression.

To further confirm the allelism between the *mc-vin* and *mc* mutants, a complementation test was carried out by crossing *mc-vin* mutants as female parent with *mc* mutant plants. All of the F1 plants produced reverted inflorescences and flowers with leafy-like sepals as their parents (Supplementary Fig. S1), which confirmed *mc-vin* as a new allele of the *MC* gene. In order to better characterize the function of *MC* in the maintenance of the IM identity, inflorescence apices from WT and the *mc-vin* mutant were compared to determine changes in gene expression using microarray analysis. A total of 999 transcripts showed altered expression levels in *mc-vin* mutant compared to WT; 482 transcripts were up-regulated (Supplementary Table S1) while 517 transcripts were down-regulated (Supplementary Table S2). As was expected, the greatest change in expression levels occurred for the *MC* gene (Logarithmic Fold Change = −4.67). The expression of several significantly up- and down-regulated genes in the *mc-vin* mutant were validated by qRT-PCR analysis as an independent control of the microarray results. Differentially up-regulated and down-regulated expressed genes in the *mc-vin* mutant relative to the WT were distributed in different Gene Ontology (GO) classes under biological processes and molecular functions categories. The main pathway regulated by *MC* was related to response to stimulus and stress, as well as primary metabolic processes (Supplementary Fig. S2A). Under the molecular functions category, most up- and down-regulated genes were involved in catalytic and binding activities, respectively (Supplementary Fig. S2B). No specific patterns of biological processes could be discerned between up- or down-regulated genes (Supplementary Fig. S2A). However, among genes with assigned molecular functions, genes associated with binding activity accounted for 62.9% of down-regulated genes in *mc-vin* compared to WT (Supplementary Fig. S2B) consistent with the suggestion that *MC* positively regulates the transcription of other genes involved in binding functions.

### *mc-vin:sft* and *mc-vin:j* double mutants phenotypic characterization

**Figure 4:**
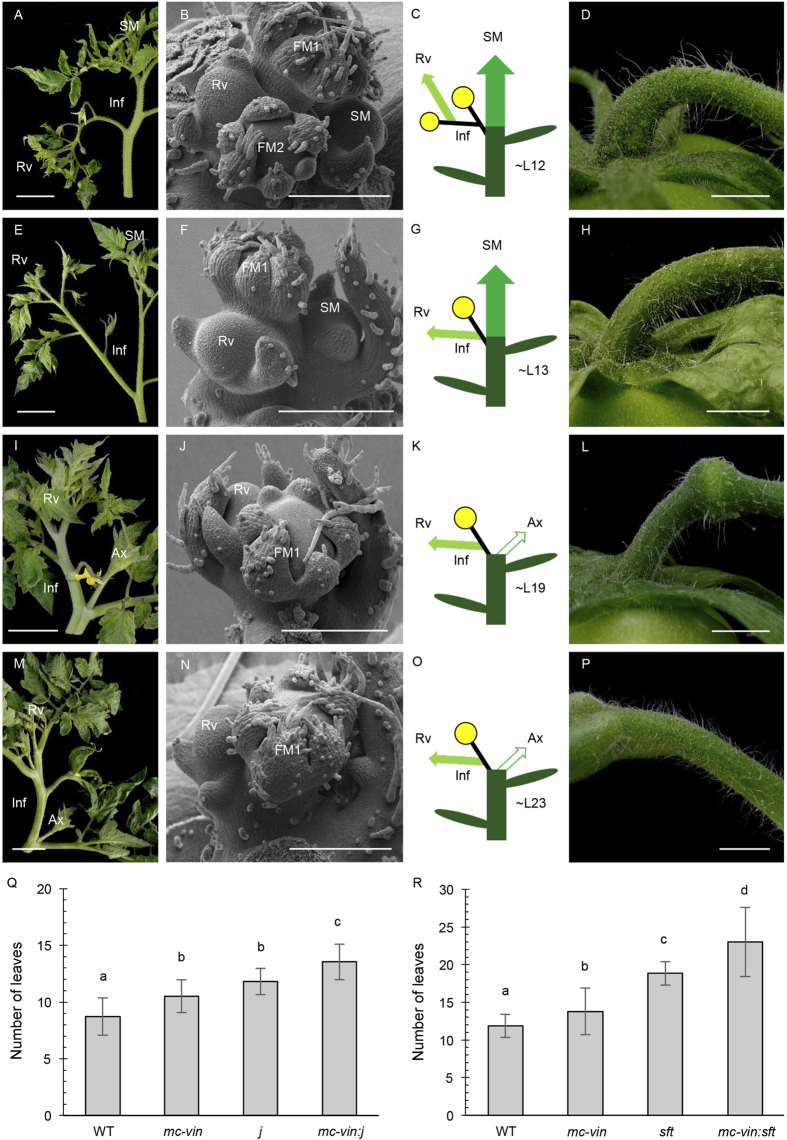
Phenotypic characterization of the *vegetative inflorescence: jointless (mc-vin:j)* and *vegetative inflorescence: single flower truss (mc-vin:sft)* double mutants in tomato. (**A**–**D**) *jointless (j) mutant*, (**E**–**H**) *mc-vin:j* double mutant. (**I**–**L**) *single flower truss (sft)* mutant. (**M**–**P**) *mc-vin:sft* double mutant. (A, E, I, M) apical shoot showing one inflorescence (Inf) reverting to vegetative growth (Rv) after development of (**A**) three flowers in j, (**E**) one flower in *mc-vin:j*, (**I**) one flower in *sft* and (**M**) one flower in *mc-vin:sft*. Note the normal development of the sympodial shoot (SM) in the (**A**) *j* and (**E**) *mc-vin:j* mutants. The SM growth is disrupted in the (**I**) *sft* and (**M**) *mc-vin:sft* mutants and it may develop as an axillary shoot (Ax). (**B**,**F**,**J**,**N**) Scanning electron microscopy (SEM) images of inflorescences containing one flower bud (in *mc-vin:j, sft and mc-vin:sft*, FM1) or two flowers buds (in j, FM1 to FM2) before initiating a reverted vegetative meristem (Rv). Note the development of the sympodial meristem (SM) at the axil of the last formed leaf in the (**B**) *j* and (**F**) *mc-vin:j* mutants and its absence in the (**J**) *sft* and (**N**) *mc-vin:sft* mutants. (**C**,**G**,**K**,**O**) Diagrams of plant development showing one inflorescence (Inf) and sympodial development (SM) if any (yellow closed circles: flowers, central column: main shoot composed of different sympodial segments, green ovals: leaves, small light green arrow: reversed vegetative growth inside the inflorescence (Rv), white arrow: disrupted SM development that grows as a regular axillary shoot (Ax)). (**D**,**H**,**L**,**P**) Fruit pedicels showing absence of abscission zone in the (**D**) *j*, (H) *mc-vin:j*, and (P) *mc-vin:sft* mutants, incomplete abscission zone in (**L**) *sft* mutant. Scale bars: 5 cm in A, E, I, and M; 1 cm in D, H, L, and O; and 300 μm in (**B**,**F**,**J**,**N**,**Q**,**R**) Flowering time of the initial segment in the F2 populations of the crosses between (**Q**) *mc-vin* and *j* mutants or (**R**) *mc-vin* and *sft* mutants. Values followed by a same letter (a, b, c or d) are not statistically different (P < 0.01).

The *SFT* and *J* genes have been reported as regulators of tomato inflorescence architecture through the control of the maintenance of IM identity^15^,^20^,^21^,^30^. Indeed, *j* and *sft* mutants produce inflorescences that revert to a vegetative growth after the initiation of few flowers (Fig. 4A–D,I–L). Tomato sympodial growth is also disrupted in the *sft* mutant (Fig. 4I–K) and both mutations affect the pedicel abscission zone development (Fig. 4D,L). Thus, *mc-vin:j* and *mc-vin:sft* double mutants were generated to better understand the genetic interactions among *MC, J*, and *SFT* during the inflorescence development.

The F2 population resulting from the cross between *mc-vin* and *j* displayed a 9:3:3:1 mendelian segregation (χ^2^ = 3.97, *P* = 0.26) since 65 WT, 25 *mc-vin*, 16 *j*, and 11 double mutant plants were observed. The *mc-vin:j* double mutant displayed abnormal reverted inflorescences containing flowers and leaves (Fig. 4E) as their parental lines (Fig. 1D, Fig. 4A). However, while the reversion of the inflorescence towards vegetative development in *mc-vin* and *j* mutants occurred after two or three flowers were developed (2.4 ± 0.3 and 2.7 ± 0.4, respectively), double mutant inflorescences reverted to a vegetative growth after the first flower (1.1 ± 0.3). Hence, the inflorescence reversion occurred earlier in the double mutant than in the *mc-vin* and *j* single mutants as only one flower was observed before the IM reverted to a vegetative meristem (Fig. 4F compared to Fig. 1K, Fig. 4B). As observed for the parental mutant plants, such an identity change of IM did not modify the sympodial growth habit of the *mc-vin:j* double mutants (Fig. 4E–G compared to Figs 1B,K–L and 4A–C). The *mc-vin:j* double mutant flowers showed leafy-like sepals (Fig. 4E,H), as occurred in the *mc-vin* mutant (Fig. 1D,H), and lacked the pedicel abscission zone (Fig. 4H), as happened in the *j* mutant (Fig. 4D). Regarding the flowering time of the initial segment, that was assessed as the number of leaves before flowering, both *mc-vin* (10.6 ± 1.4) and *j* (11.8 ± 1.2) single mutants showed a significant delay in flowering compared to WT plants (8.7 ± 1.7), whereas the *mc-vin:j* double mutant flowered somewhat later (13.5 ± 1.6) than its parental mutants (Fig. 4Q), suggesting a synergistic interaction between *MC* and *J* to control the timing of floral transition.

The *mc-vin* mutant was also crossed with *sft,* and a phenotypic analysis was performed on 80 F2 plants, of which 49 WT, 15 *mc-vin,* 13 *sft*, and 3 double mutant plants were observed. Thus, the F2 population displayed a 9:3:3:1 Mendelian segregation (χ^2^ = 1.42, *P* = 0.7). The *sft* mutant developed one to three flowers before the IM reverted to a vegetative growth (an average of 1.7 ± 0.9), whereas the reversion of the inflorescence towards vegetative development in *mc-vin* was found after producing at least two flowers (2.6 ± 0.4). Combination of *mc-vin* and *sft* mutations resulted in plants with a terminal inflorescence that reverted to vegetative growth after the initiation of a single flower (1.2 ± 0.4), which also developed leafy sepals (Fig. 4M). As occurred in *sft* mutant plants (Fig. 4J), SM of *mc-vin:sft* plants did not develop from the axil of the last formed leaf (Fig. 4N). The disruption of sympodial growth and inflorescence development (Fig. 4O) are reminiscent of the single *sft* mutant phenotype (Fig. 4I,K) although inflorescence phenotype was much more robust in the double *mc-vin:sft* mutant since inflorescences with more than one flower before reversion were never observed. Regarding flower development, the leafy character of the sepals was slightly stronger in the *mc-vin:sft* double mutant (Fig. 4M) than in the *mc-vin* single mutant (Fig. 1D) and the double mutant flower completely lost pedicel abscission zone (Fig. 4P) while it is incomplete but still present in the *sft* (Fig. 4L) and *mc-vin* (Fig. 1F) single mutants. Respect to flowering time, pairwise comparisons of means using least significant difference (LSD) test showed significant differences (*P *< 0.05) between *mc-vin* (13.4 ± 3.3 leaves) and *sft* (18.9 ± 1.6 leaves) mutant plants for this trait. Moreover, the *mc-vin:sft* double mutant plants displayed a more severe delay in flowering (23.0 ± 4.6 leaves) compared to the single mutant parents (Fig. 4R).

### Expression of *MC* in *mc-vin, j* and *sft* mutants

**Figure 5:**
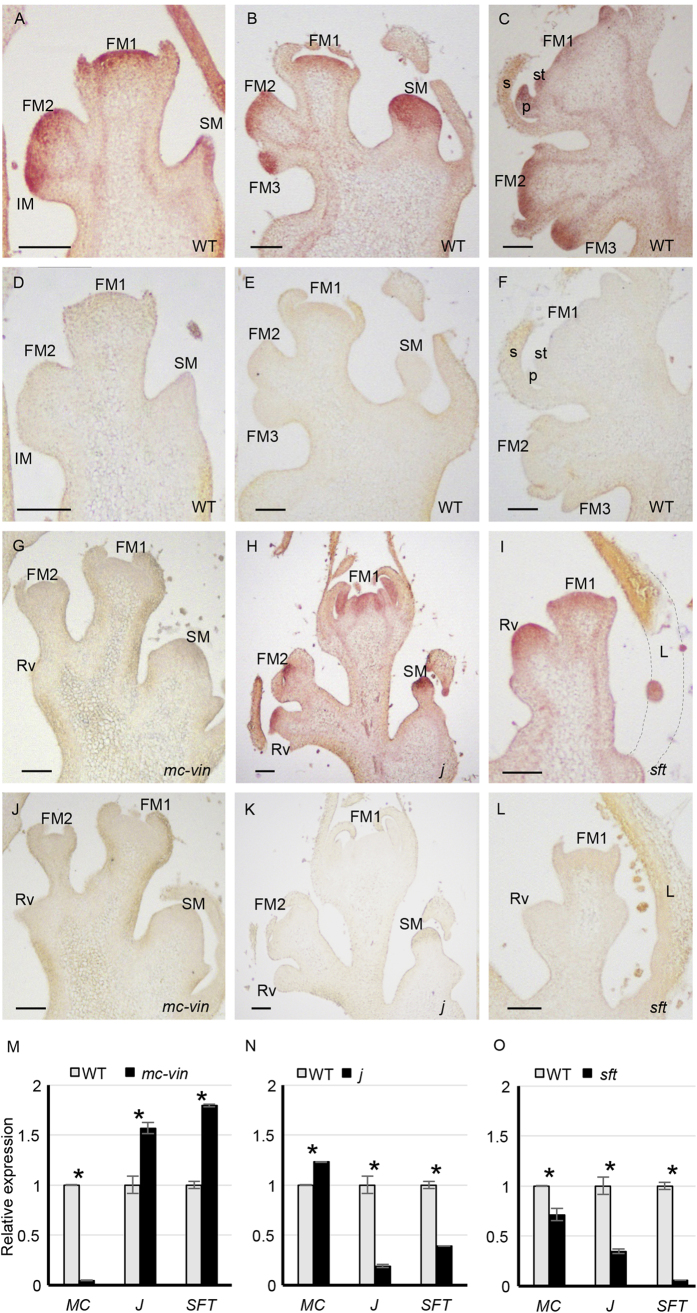
Tissue localisation of *MACROCALYX* (*MC*) transcripts by means of *in situ* hybridisation in inflorescences of (**A–F**) the wild type Moneymaker (WT) cultivar, (**G,J**) the *vegetative inflorescence* (*mc-vin*) mutant, (**H,K**) the *jointless* (*j*) mutant and (**I,L**) the *single flower truss* (*sft*) mutant. (**A**,**B**,**C**,**G**,**H**,**I**) *MC* antisense probe; (**D**,**E**,**F**,**J**,**K**,**L**) *MC* sense probe used as negative control. FM, floral meristem; IM, inflorescence meristem; Rv, reverted vegetative meristem; SM, sympodial meristem; L, leaf; s, sepal; p, petal; st, stamen. Scale bars: 100 μm. (**M**,**N**,**O**) Relative quantitative RT-PCR expression analysis of *MACROCALYX (MC)* in flowering shoot apices of (M) the *vegetative inflorescence* (*mc-vin*) mutant, (**N**) the *jointless* (***j***) mutant and (**O**) the *single flower truss* (*sft*) mutant compared to wild type Moneymaker (WT) cultivar. ns, no statistically significant differences; *significant differences at *P* < 0.05.

To further investigate the interactions among *MC, J* and *SFT* in the inflorescence development, *MC* expression in inflorescence buds of *mc-vin, j* and *sft* plants was analysed by *in situ* hybridization experiments. The *MC* gene was strongly expressed in IM, FM and young flower buds of WT inflorescences as well as in SM and the leaf subtending it (Fig. 5A–C). During the first stages of flower morphogenesis, *MC* transcripts were strongly accumulated in the centre of the flower bud and at a lower level in the sepal primordia (Fig. 5A). Later, *MC* expression disappeared in young sepals and was detected in the petal and stamen primordia as well as in the innermost carpel meristematic tissue (Fig. 5C). No *MC* expression was observed in the vegetative meristem resulting from the reversion of IM nor in FM and floral buds of the *mc-vin* inflorescence apex (Fig. 5G). Contrarily, *MC* transcripts were detected in both FM and floral buds of *j* and *sft* inflorescences, and also in the reverted IM meristem (Fig. 5H,I). Regarding *MC* expression in the SM, this was not detected in *mc-vin* mutant plants but was maintained in *j* mutant plants at a similar level than in WT (Fig. 5A,B,H). In *j* and *sft* mutants, *MC* transcripts were also detected in the vascular bundles at a lower extent than observed in the WT (Fig. 5A–C,H,I).

In addition, the expression of *MC*, *J* and *SFT* was also investigated by qRT-PCR in WT, *mc-vin, j* and *sft* flowering shoot apices containing young inflorescence shoots. In the *mc-vin* mutant, *MC* expression was severely silenced and the expression of *J* and *SFT* was significantly increased compared to WT (Fig. 5M), which agreed with the results of *in situ* hybridization experiments. The *MC* transcript level slightly increased in *j* and decreased in *sft,* while both *J* and *SFT* expression levels decreased significantly in *j* and *sft* mutants, respectively, compared to WT (Fig. 5N,O).

## Discussion

Genetic and molecular analyses of the *mc-vin* mutation revealed that this corresponds to a new allele of the *MC* MADS-box gene. The role of *MC* in regulating sepal identity and pedicel abscission zone development was previously reported^23^,^24^,^25^,^31^. However, how *MC* contributes to the control of IM fate remains poorly understood. It was previously hypothesized that J could interact with a MADS-box protein induced by systemic SFT protein to prevent vegetative growth in the IM^8^,^11^. The MC protein could somehow play a role in this complex. Indeed, the phenotypes of *mc-vin* and *mc* inflorescence resemble those shown by *j* and *sft* mutants, as they displayed reversion of the inflorescence towards vegetative development suggesting that *MC, J* and *SFT* may interact to control IM fate in tomato inflorescence. Results showed that the reversion of inflorescence towards vegetative development occurred earlier in both *mc-vin:sft* and *mc-vin:j* double mutants than in the single mutants, which indicates that *MC* synergistically interacts with *SFT* and *J* to control inflorescence architecture of tomato (Table 1). In addition, the *j:sft* double mutant has been previously described and a synergistic interaction between *SFT* and *J* has been reported^11^. These three genes have, thus, not completely overlapping functions and may cooperate to confer IM identity to new meristems that are formed after floral transition of the SAM.

Both *J* (reported in^11^,^30^) and *MC* (this work) are expressed in IM while *SFT* is mainly expressed in the leaves and encodes a systemic signal^21^. Nonetheless, it has been reported that *SFT* transcripts accumulate during tomato meristem maturation^10^. Therefore, an increased *SFT* expression found in IM might have a repressing effect on genes integrating the vegetative developmental program. MC and J were previously shown to be able to form a MADS-box heterodimer^24^ and to be part of a complex controlling the pedicel abscission zone development^25^,^26^. The co-localization of *J* and *MC* expression in IM suggests that such a MC/J complex could also be involved in IM fate control. The involvement of MC in protein complexes is further supported by microarrays results here reported (Supplementary Fig. S2B), which showed that *MC* positively regulates the expression of several genes involved in binding function. Gene expression analysis indicated moreover that *MC* could partly repress *J* and *SFT* while *J* and *SFT* partly activate each other’s expression in the flowering shoot apex. It is reasonable to think that *SFT* and *J* are involved in a positive feedback loop, which in turn could be repressed by *MC*. In *Arabidopsis*, the *MC* homologue *AP1* participates in different regulatory loops to fine-tune floral initiation in concert with *FT* and *SVP/AGL24*, the homologues of *SFT* and *J* respectively^32^,^33^. In FM, SVP and AGL24 directly interact with AP1 in a complex to repress *TFL1* in young FM, while shortly after the onset of flower formation, *AGL24* and *SVP* are down-regulated by *AP1* to appropriate levels to prevent reversion of FM into vegetative shoot structures^32^,^34^. Additional actors are involved in these regulatory loops. It is tempting to propose that a similar interconnected pathway as observed in the FM in *Arabidopsis* is involved in the regulation of IM fate in tomato. The low down- and up-regulations observed between the investigated genes in this work suggested that other factors could be involved in addition to *MC, SFT* and *J* to control IM fate in tomato.

Most of the genes involved in inflorescence development also regulate flowering time in tomato (reviewed in^5^,^6^,^7^). Accordingly, a new function for *MC* was identified as floral activator since the *mc-vin* mutant has a late flowering character. A role for *MC* in the control of flowering time was not reported before, as far as this study is concerned. However, a role for *AP1* homologue in tomato as floral promoter has been suggested from the early flowering phenotype displayed by transgenic tomato plants overexpressing the *Arabidopsis AP1* gene^35^. Such an overexpression of *AP1* or its homologues also caused early flowering in different monocot and dicot species^36^, confirming a shared role of *AP1* homologues in flowering time control of angiosperms.

The double mutant characterization in this work revealed that *MC* synergistically interacts with *J* and *SFT* to control the timing of floral transition in tomato (Table 1). Indeed, both *mc-vin:j* and *mc-vin:sft* double mutants have a late flowering phenotype that is increased compared to their parental mutants. The involvement of *J* and *SFT* in floral promotion was previously reported^15^,^20^,^21^. The *J* gene usually interacts with genes controlling flowering time, even though *SFT* is epistatic to *J* regarding floral transition timing^11^,^37^. All double mutants reported so far having *sft* as parent show late flowering or are unable to do so^11^,^20^,^21^ which highlights the key role of *SFT* in the flowering time regulation in tomato. *SFT* encodes a florigen, and triggers graft-transmissible signals that induce flowering^21^,^22^. Therefore, when SFT reaches the shoot apex, *J* and *MC* could interact with it to activate floral transition in a similar way as they cooperate to control IM fate.

Tomato has a sympodial growth pattern, thus floral transition occurs more than once during the plant’s life. The sympodial growth is disrupted in both the *sft* single mutant^20^,^21^ and the *mc-vin:sft* double mutant, but not in the *mc-vin* single mutant, which indicates an epistatic effect of *SFT* on *MC* regarding sympodial growth (Table 1). *SFT* was previously reported to be required for proper sympodial growth development in tomato^20^,^22^,^31^. However, *MC* and *J* are not involved in the control of this developmental process since sympodial development was maintained in *mc-vin* and *j* mutants as well as in the *mc-vin:j* double mutant. All three mutants used to produce three leaves in the sympodial segments before floral transition as observed in the wild type (data not shown). These observations showed that although *MC* and *J* control the timing of floral transition in the initial segment, they do not control the flowering time of the sympodial segments, suggesting that floral transition is not regulated by the same genes in the initial and sympodial segments of tomato plants.

One of the most notable traits of the *mc-vin* phenotype is the conversion of sepals into leaf-like organs. The *MC* gene is thus required for sepal identity and plays a role as A class organ identity gene like *AP1* in *Arabidopsis* and *SQUAMOSA* in *Antirrhinum*^23^. However, *MC* only controls sepal identity as does *SQUAMOSA,* while *AP1* controls both sepal and petal identity^38^,^39^. The conversion of sepals into leafy structures could be explained by the loss of floral organ identity in the *mc-vin* first floral whorl. Indeed, in *Arabidopsis,* the loss of *AP1* results notably in the formation of bracts instead of sepals in the first floral whorl, and complete loss of the floral organ identity in the class E quadruple mutant *sep1:sep2:sep3:sep4* leads to conversion of all floral organs into leaf-like organs^40^. Inside the flower, *MC* expression is not restricted to the first floral organ whorl; therefore, *MC* could play a broader role during tomato flower development as observed for *AP1*. In *Arabidopsis*, *AP1* establishes floral meristem identity and coordinates the formation of floral primordia by regulating genes involved in organ growth and patterning before initiating downstream pathways required for floral organ specification^32^. The precise functional role of *MC* during flower development of tomato would need further research.

Regarding sepal development, it was shown that *MC* synergistically interacts with *SFT* (Table 1), given the stronger leafy sepal development in the *mc-vin:sft* double mutant compared to the *mc-vin* single mutant. However, overexpression of *SFT* does not suppress the leafy-sepal development of the *mc* mutant flowers (Shalit *et al.* 2009), proving that the loss of sepal identity cannot be overcome by *SFT*. Furthermore, *SFT* was shown to be involved in leaf development, regulating leaf architecture^31^. In the *mc-vin:sft* double mutant, the mutation in *MC* might be responsible for the formation of a leafy sepal and the *sft* mutation may only reinforce its leafy identity. According to this study’s results, *J* seems not to be involved in sepal development, and *MC* is epistatic to *J* regarding this trait. However, combining *j* mutation with other mutations such as *sft* and mainly *blind* resulted in flowers bearing sepals with a more or less strong leafy appearance^11^,^30^ suggesting that *J* could also have a role in sepal identity. It could not be excluded that *MC* is required to maintain sepal identity in the first floral whorl, and when it is lost such as occurred in the *mc-vin* mutant, the absence of *SFT* and *J* functions strengthens the vegetative character of the developing sepals.

## Methods

### Plant material and phenotypic analysis

The *mc-vin* T-DNA mutant was isolated from a collection of T-DNA insertion lines generated from seeds of cv. Moneymaker (MM). The genetic transformation was performed according to our previous protocol^41^ via *Agrobacterium* strain LBA4404 containing the enhancer trap vector pD991^27^ (kindly supplied by Dr. Thomas Jack; Department of Biological Sciences, Dartmouth College, USA). Due to the recessive nature of the *mc-vin* T-DNA mutant, it was selected for its phenotype (reversion of inflorescence meristems to vegetative growth after forming two or three flowers with leaf-like sepals) from a T2 segregating line. The *mc-vin* mutant was used as female parent in crosses with *sft* and *j* mutants to generate *mc-vin:sft* and *mc-vin:j* double mutants respectively. In addition, a complementation test was carried out by crossing *mc-vin* mutants as female parent with *mc* mutant plants. Seeds of the *sft* (accession number LA2460, background Platense), *j* (accession number LA3033, background Gardener), and *mc* (accession number LA0159, background unknown) mutants were obtained from the Tomato Genetics Resource Center (http://tgrc.ucdavis.edu/). Plants were grown under greenhouse conditions using standard practices with regular addition of fertilizers.

The flowering time of the initial segment was assessed as the number of leaves produced below the first inflorescence. Additionally, the number of flowers was recorded for the two first inflorescences of each genotype. Segregation ratio in F2 populations was analysed by χ^2^ test for goodness-of-fit to the expected ratio 9:3:3:1. The least significant difference (LSD) test (SAS Institute, Carry, NC, USA) was used to compare the mean values. A probability of *P* < 0.01 was considered statistically significant.

### Scanning-electron microscopy (SEM)

SEM studies were performed as described^42^. Plant material was fixed in FAEG (10% formaldehyde, 5% acetic acid, 50% absolute ethanol, and 0.72% glutaraldehyde) and stored in 70% ethanol. The samples were dehydrated, critical point dried with liquid CO_2_ in a critical point dryer Bal-Tec CPD 030 and gold coated in a Sputter Coater (Bal-Tec SCD005). The samples were visualized with a Hitachi S-3500N scanning electron microscope at 10 kV.

### DNA isolation

Tomato genomic DNA was isolated as described^43^. Genomic DNA was quantified by fluorometry using SYBR Green I (Sigma-Aldrich) as fluorophore. Fluorescence measurements were made at room temperature using Synergy MX (Biotek) fluorometer.

### Southern blot analysis

In order to determine the number of T-DNA insertions existing in the *mc-vin* mutant, a DNA-blot hybridization was performed from 10 μg of genomic DNA digested by restriction enzymes *Eco*RI and *Hind*III, electrophoresed throughout 0.8% agarose gel, and blotted onto Hybond N+ membranes (GE Healthcare). Hybridization was carried out as described^44^ with a chimeric probe, fusing the complete coding sequence of the *NEOMYCIN PHOSPHOTRANSFERASE II* (*NPTII*) gene to 811 pb of coding sequence of endogenous tomato *FALSIFLORA* (*FA*) gene, which was employed as hybridization positive control. Finally, the chimeric probe was labelled with [α-^32^P]dCTP using High Prime random priming kit (Roche Applied Science) following manufacturer’s instructions. Nylon membranes were exposed to Hyperfilms (GE Healthcare).

### Identification of sequences flanking T-DNA insertion sites

The sequences flanking T-DNA were isolated by Anchor-PCR according to the procedure previously established^28^,^29^. The sequence of primers used is listed in Supplementary Table S3. The cloned sequences were compared with SGN Database (http://solgenomics.net/) to assign the T-DNA insertion site on tomato genome.

### PCR Genotyping

Co-segregation of the T-DNA insertion site with the mutant phenotype in the T2 progeny was checked by PCR using i) the specific genomic forward (Genotyping-F) and reverse (Genotyping-R) primers to amplify the WT allele (without T-DNA insertion) and ii) one specific genomic primer (Genotyping-F) and the specific T-DNA border primer (T-DNA-R) to amplify the mutant allele (carrying the T-DNA insertion). The primers located upstream and downstream of the T-DNA insertional sites in each line were designed based on sequence information available from SGN Database (http://solgenomics.net/). The sequence of genotyping primers used is listed in Supplementary Table S3. Amplification of genotyping primers was performed in a volume of 30 μl using 25 ng of total DNA, 50 ng of each primer, 0.25 mM dNTPs, 2.5 mM MgCl_2_, and 1 U of REDTaq DNA polymerase (SIGMA-Aldrich) in 1X Taq buffer. DNA was amplified under the following thermal cycling conditions: 94 °C for 5 min, followed by 35 cycles at 94 °C for 30 s, 60 °C for 30 s, and 72 °C for 2 min, and a final extension of 5 min at 72 °C. PCR products were analysed in 1% agarose gels in SB buffer (10 mM sodium boric acid) and visualized with ethidium bromide.

### RNA Isolation

Total RNA was isolated using TRIZOL (Invitrogen) according to the manufacturer’s instructions. Contaminating DNA was removed using the DNA-free^TM^ kit (Ambion). RNA quantity and quality were assessed by gel electrophoresis and spectrophotometry (GeneQuant II, Pharmacia Biotech).

### Quantitative Real Time PCR (qRT-PCR) analysis

A total of 500 ng of RNA was used for cDNA synthesis using M-MuLV reverse transcriptase (Fermentas Life Sciences) as well as a mixture of random hexamer and oligo(dT)_18_ primers. Specific primer pairs for each gene (Supplementary Table S3) were used for expression analysis by qRT-PCR performed with the SYBR Green PCR Master Mix (Applied Biosystems) kit using the 7300 Real-Time PCR System (Applied Biosystems). Data collection and analysis were performed using 7300 System Sequence Detection Software v1.2 (Applied Biosystems). Results were expressed using the ∆∆Ct calculation method in arbitrary units by comparison to a data point from the wild type samples. The housekeeping gene *Ubiquitine3* (Solyc01g056940.2.1) was used as a control in all gene expression analyses. The significance of pairwise comparisons between genotypes was assessed by using LSD test (*P*<0.05). The absence of genomic DNA contamination in the qRT-PCR assays was evaluated using a tomato-specific amplicon (intron sequence) as control.

### Microarray Experiments

The transcriptomic study of *S. lycopersicum* gene expression between WT and *mc-vin* T-DNA mutant inflorescence apices was done using an Agilent Tomato Oligo Microarray containing 60,000 probes. Three independent biological replicates were performed. Hybridization targets were prepared according to Agilent’s Two-Color Microarray-Based Gene Expression Analysis protocol v.6.5 (Agilent). Microarray slides were scanned using the Agilent Technology Microarray Scanner. Spot signal intensities were acquired using Feature Extraction v.10.7 software (Agilent). Inter-array normalization was done using the quantiles method^45^, which was implemented in R and included in the Bioconductor package^46^. Statistical analysis was conducted using the Bioconductor packages Limma, Marray, PCAmethods, and EMA. The unpaired *t* test assuming equal variances was used for statistical comparison between the different data sets. A gene was considered differentially expressed when the absolute log Fold-Change was > 0.3 and the corresponding adjusted P-value < 0.05. The expression of several significantly up- and down-regulated genes in the *mc-vin* mutant was validated by qRT-PCR analysis as an independent control of the microarray results (Supplementary Fig. S3). For the functional classification of each sequence, GO annotation search was performed at The Arabidopsis Information Resource (TAIR, http://arabidopsis.org/).

### *In situ* hybridization

For *in situ* hybridization experiments, tissue preparation, sectioning and transcript detection were performed as described^42^. A *MC* probe was prepared using cDNA as template (393-pb fragment of Solyc05g056620.1.1) and excluding the MADS-box domain. Antisense transcripts were synthesized using the DIG RNA labelling mix (Roche Applied Science). As negative control, sense RNA probes were hybridized with the same sections and no signals were observed under the hybridization and detection conditions used.

## Additional Information

**How to cite this article**: Yuste-Lisbona, F. J. *et al.* Characterization of *vegetative inflorescence* (*mc-vin*) mutant provides new insight into the role of *MACROCALYX* in regulating inflorescence development of tomato. *Sci. Rep.* **6**, 18796; doi: 10.1038/srep18796 (2016).

## Supplementary Material

Supplementary Information

## Figures and Tables

**Table 1:** Tomato double mutants produced and analysed in this study.

Interaction	*mc-vin:j*	*mc-vin:sft*
Flowering time	synergistic	synergistic
Sympodial growth	no interaction	*SFT* epistatic
Inflorescence	synergistic	synergistic
Sepals	*MC* epistatic	synergistic
Abscission zone	*J* epistatic	synergistic
