# Supplementary material for: Characterization of vegetative inflorescence (mc-vin) mutant provides new insight into the role of MACROCALYX in regulating inflorescence development of tomato
Source: Sci Rep. 2016 Jan 4;6:18796. doi: 10.1038/srep18796 (PMC4698712; doi:10.1038/srep18796)
Supplement: Supplementary Information [file srep18796-s1.pdf]

Characterization of *vegetative inflorescence* (*mc-vin*) mutant provides new insight into the role of *MACROCALYX* in regulating inflorescence development of tomato

Fernando J. Yuste-Lisbona[1,+], Muriel Quinet[1,+,#], Antonia Fernández-Lozano[1], Benito Pineda[2], Vicente Moreno[2], Trinidad Angosto[1], Rafael Lozano[1,*]

[1] Centro de Investigación en Biotecnología Agroalimentaria (BITAL), Universidad de Almería, 04120 Almería, Spain.

[2] Instituto de Biología Molecular y Celular de Plantas (UPV-CSIC), Universidad Politécnica de Valencia. Avenida de los Naranjos s/n. 46022 Valencia, Spain

[#] Present address: Groupe de Recherche en Physiologie végétale, Earth and Life Institute, Université catholique de Louvain, Croix du Sud 4-5 bte L7.07.13, B-1348 Louvain-la-Neuve, Belgium

[+] These authors contributed equally to this work

[*] Corresponding author: rlozano@ual.es

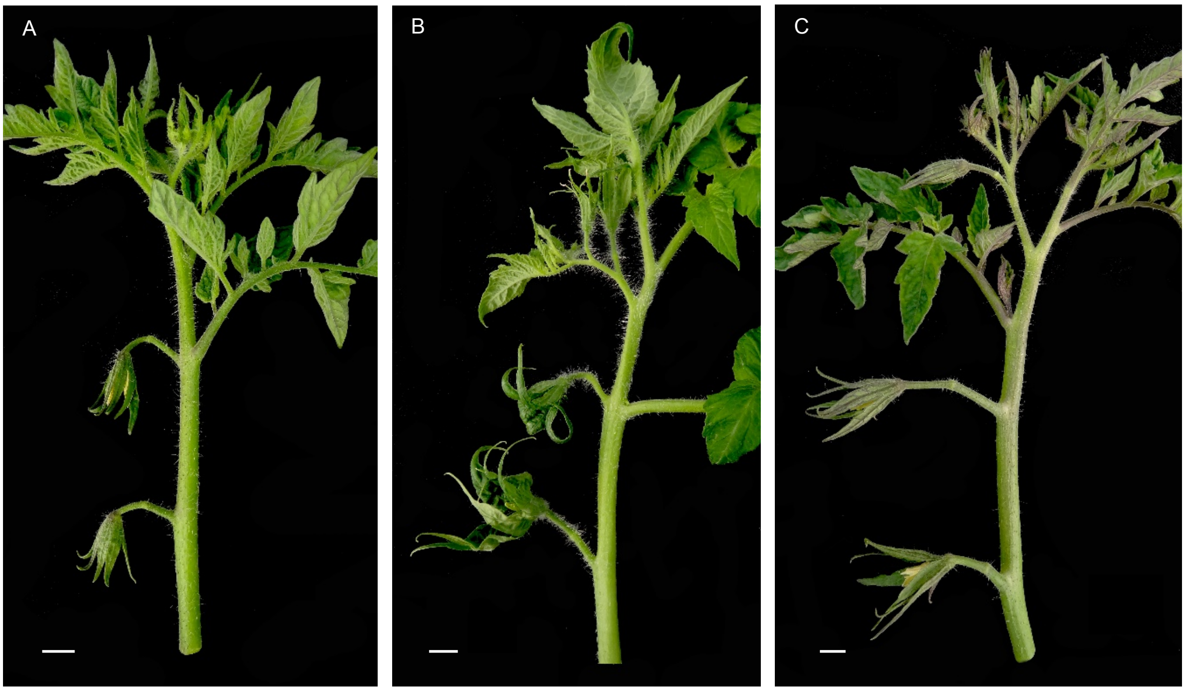

Supplementary Figure S1. Complementation test between *mc-vin* and *mc* (accession number LA2460) mutant plants. Complementation test was carried out by crossing a *mc-vin* mutant as female parent (A) with a *mc* mutant plant (B). All of the F1 plants produced reverted inflorescences and flowers with leafy-like sepals (C). Scale bars: 1 cm.

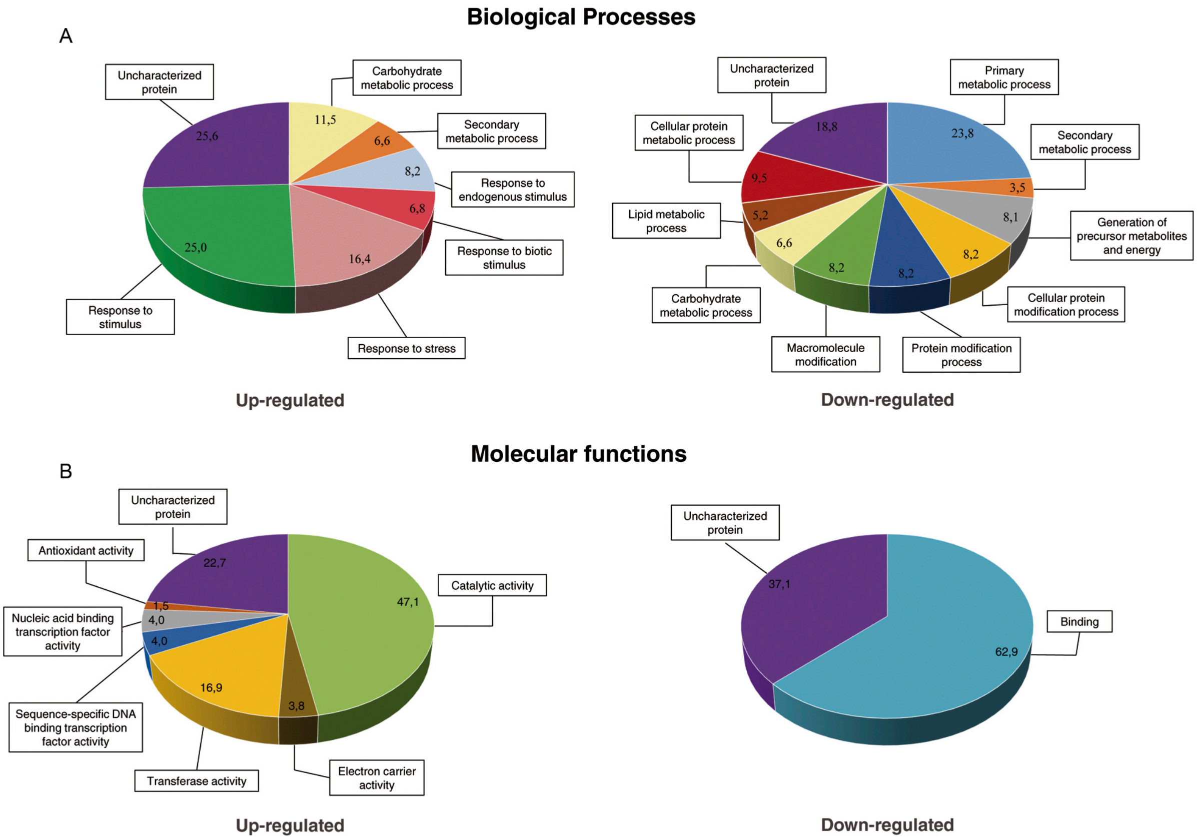

Supplementary Figure S2. Distribution of differentially up-regulated and down-regulated expressed genes in the *mc-vin* mutant relative to the wild-type Moneymaker cultivar in Gene Ontology (GO) annotation under (A) biological processes and (B) molecular functions categories.

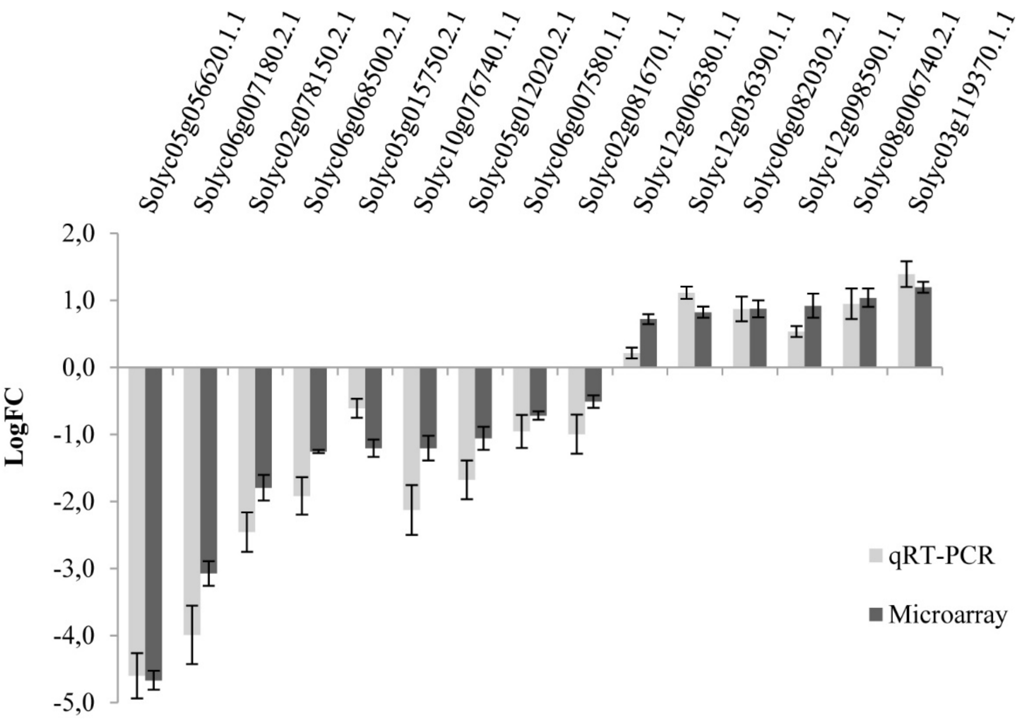

Supplementary Figure S3. Validation of the gene expression data obtained by the microarray using qRT-PCR. The panel show a comparison of the LogFC in expression levels measured by the microarray and qRT-PCR assays of 15 differentially expressed genes in the *mc-vin* mutant relative to the wild-type Moneymaker cultivar chosen at random from the microarray data.

Supplementary Table S1. Differentially up-regulated expressed genes in the *vegetative inflorescence* (*mc-vin)* mutant relative to the wild-type Moneymaker cultivar (adjusted p-value <0.05)

GenBank Accession	TIGR Tomato Gene Index	SGN gene model ID	Gene Description	LogFC	adjusted p-value
AW091778			uncharacterized protein	1.8177528	0.00234593
AK320517	TC243427		glutathione s-transferase	1.75779773	0.03911017
AK323458	TC239836		auxin-induced protein	1.55459005	0.00107071
AW036494			uncharacterized protein	1.27643791	0.00983314
AK328854	TC223575		uncharacterized protein	1.26638447	0.0391856
AI490010		Solyc03g119370.1.1	dna binding	1.19300129	0.03042268
AK323953	TC226520	Solyc06g060760.2.1	aquaporin tip2-3	1.178691	0.02736339
XR_182885		Solyc06g062380.2.1	acid phosphatase	1.13441026	0.03241277
AK321469	TC231875		membrane protein	1.13376524	0.01216619
DV103959			heat stress transcription factor a-6b-like	1.12440262	0.02933588
FS199047			uncharacterized protein	1.11323542	0.00107071
AK329386	TC225826	Solyc11g006910.1.1	ferredoxin- chloroplast	1.10507625	0.00550285
AK323732	TC220284	Solyc06g062370.2.1	stem 28 kda glycoprotein	1.1023103	0.03034746
AW216944		Solyc04g054690.2.1	l-ascorbate oxidase	1.06959239	0.02660843
DV104735			uncharacterized protein	1.04560703	0.02475616
AK323831	TC218686	Solyc08g006740.2.1	histidine decarboxylase	1.03749797	0.01111077
AI896114		Solyc02g068470.1.1	uncharacterized protein	1.0325516	0.0313441
AK326930	TC217220	Solyc04g054690.2.1	l-ascorbate oxidase	1.00544205	0.01923255
AW617236			transposase	0.99952065	0.00529177
BW691204			uncharacterized protein	0.97251921	0.02533161
AI778224			glutathione s-transferase	0.97205833	0.01175905
EG553019	TC233327		uncharacterized protein	0.95665028	0.03645518
AK328082	TC232716	Solyc10g085230.1.1	uncharacterized protein	0.95148919	0.00757321
AK322756			cytochrome p450	0.9394876	0.04726932
AK329756	TC217479		regulator of vps4 activity in the mvb pathway protein	0.93868266	0.01513849
Z18277	NP000412	Solyc02g085020.2.1	dihydroflavonol 4-reductase	0.93356648	0.0136293
AJ785277			iaa-amino acid hydrolase 9	0.93348316	0.00784769
AW035871		Solyc01g103630.2.1	lactoylglutathione lyase	0.93328354	0.03312049
BT014540	TC218176	Solyc12g009930.1.1	zeatin o-glucosyltransferase-like	0.92899689	0.01922578
AK319507	TC218425	Solyc12g057070.1.1	udp-glycosyltransferase 85a2-like	0.9242709	0.03791821
DB710746	TC221366	Solyc08g068600.2.1	histidine decarboxylase	0.92417758	0.02178015
AW032620			uncharacterized protein	0.91837343	0.0323025
AK324517		Solyc12g098590.1.1	udp-glycosyltransferase 75d1-like	0.91836888	0.01129466
AJ278765	TC217814		sugar transporter erd6-like 7	0.91768151	0.00550285
AK326564	TC217475		glycine-rich protein	0.91108938	0.03503395
AK328581	TC228944		auxin-induced protein	0.89894606	0.03510861
AK325240	TC228215		cxe carboxylesterase	0.89500475	0.04830703
BT013539	TC217273		beta-glucosidase 44-like	0.89499028	0.00529177
BG130930			alpha-l-fucosidase 2	0.89219817	0.0136293
AK328389	TC242265	Solyc10g085010.1.1	uncharacterized protein	0.88408489	0.03213262
BP894990			uncharacterized protein	0.874999	0.01112478
CK720539		Solyc06g082030.2.1	gibberellin 20-	0.87194341	0.01129466
ES894405			s-adenosyl-l-methionine:benzoic acid salicylic acid carboxyl methyltransferase	0.86959308	0.02973177
AJ784587	TC238182	Solyc07g008380.1.1	uncharacterized protein	0.8545307	0.01824748

AK327911			uncharacterized protein	0.85327727	0.04915587
AW039501			uncharacterized protein	0.85206116	0.00684964
GO373764	TC245035	Solyc02g062500.2.1	2-oxoglutarate-dependent dioxygenase	0.8511215	0.01922613
AK324883	TC217742	Solyc08g068690.1.1	tyramine hydroxycinnamoyltransferase	0.84797063	0.02545931
AK329690	TC236171		cyanidin-3-o-glucoside 2-o-glucuronosyltransferase-like	0.83831862	0.02836822
X98308	TC218993	Solyc06g053610.2.1	myb-related transcription factor	0.83637607	0.02338954
DB700199	TC242706		udp-d-glucose udp-d-galactose 4-epimerase 1	0.82374445	0.02061969
DB684305	TC229789	Solyc12g036390.1.1	calmodulin-binding protein	0.82188739	0.03820845
AK327834			uncharacterized protein	0.82175642	0.0113692
AK329308		Solyc03g044100.2.1	peroxidase 73	0.82118536	0.01430581
DB710333			nucleic acid binding	0.82071775	0.03886765
XM_004248335		Solyc10g006660.2.1	Calcium-binding EF hand family protein	0.81195343	0.03334823
BM412833	TC220122		probable anion transporter chloroplastic-like	0.80928922	0.01728035
AK322040	TC228312		uncharacterized protein	0.80344398	0.01482666
BI422664	TC236102	Solyc07g006420.1.1	uncharacterized protein	0.80109261	0.01978442
AK329230	TC221492		non-symbiotic hemoglobin class 1	0.78551911	0.02540922
AK323654	TC232885	Solyc12g008350.1.1	dehydration-responsive element-binding protein 3-like	0.7839564	0.0211868
AW033463			peroxidase	0.78203416	0.0153113
BG127002	TC219400		proline-rich protein	0.78063247	0.01570102
AK321017	TC240930		delta-type tonoplast intrinsic protein	0.77789372	0.04897428
AJ786362	NP9243938	Solyc10g086500.1.1	uncharacterized protein	0.77312611	0.00781667
EG553012	TC236685	Solyc10g007280.2.1	mitochondrial chaperone bcs1	0.77228359	0.04844452
XM_004245412	TC238487	Solyc08g083090.1.1	uncharacterized protein	0.7641747	0.00784769
AK329507	TC229571		uncharacterized protein	0.75570957	0.03880424
AK246959		Solyc08g059710.2.1	uncharacterized protein	0.74635072	0.02283711
AK247936	TC228316	Solyc08g068850.2.1	proton pump interactor 1	0.74604225	0.02660843
AK319193	TC240501		nad h dehydrogenase b2	0.73441262	0.03859621
AK324991	TC241519		cinnamoyl reductase	0.72838794	0.00931396
AI486264	TC231285	Solyc12g005430.1.1	uncharacterized protein	0.72627188	0.01513849
BT012735	TC239525		proline dehydrogenase	0.72526397	0.03518348
AW625231			histidine decarboxylase	0.72142508	0.022386
BT013817	TC237888		atp-citrate synthase beta chain protein 1-like	0.72037979	0.01061813
DV103891	NP000198	Solyc08g062450.1.1	class ii small heat shock protein le-	0.71884493	0.03042268
AK321346	TC218858	Solyc08g080170.2.1	3-hydroxy-3-methylglutaryl coenzyme a synthase	0.71873923	0.0420314
AK324589	TC232049	Solyc12g006380.1.1	desacetoxyvindoline 4-	0.71823192	0.04070823
AW622134			uncharacterized protein	0.71749367	0.02292687
BE433370			uncharacterized protein	0.71749037	0.02292687
GT166076			uncharacterized protein	0.71557649	0.0090368
AK323977	TC236975		plasma membrane intrinsic protein	0.71554159	0.04273341
XM_004251435		Solyc12g005300.1.1	chlorophyllase 2	0.71293046	0.03390315
AK246855	TC227047		hin1-like protein	0.71165418	0.04243355
AI487050		Solyc09g011860.2.1	uncharacterized protein	0.71159303	0.0113692
AW219781	TC234758	Solyc10g085280.1.1	udp-glycosyltransferase 1	0.71076	0.02545931
BI210949	TC225615	Solyc05g015850.2.1	wrky transcription	0.70898599	0.03791821
BT012795	TC229127		rubisco activase	0.7088392	0.02907994
AK328022		Solyc11g066580.1.1	flavonoid 3 -hydroxylase	0.70488776	0.03089331
AK323868	TC231135	Solyc07g043460.2.1	cytochrome p450	0.70382877	0.01129466
CK468710	TC223329	Solyc09g011590.2.1	auxin-induced protein	0.69829345	0.03135169
CK715337			uncharacterized protein	0.69692367	0.01249343
AF029349	TC217277	Solyc04g082030.1.1	ornithine decarboxylase	0.69588897	0.01940886

DV104500		Solyc09g011570.2.1	auxin-induced protein	0.6958605	0.02338426
GT165473			uncharacterized protein	0.69422717	0.01573238
AK323208	TC218910		aspartic proteinase	0.6936543	0.03014318
AW625000			uncharacterized protein	0.69361836	0.04451332
AK323147	TC239224	Solyc01g096670.2.1	cytochrome p450	0.69356616	0.03968076
BG131617	TC235620		2-oxoglutarate-dependent dioxygenase	0.69326426	0.02292687
XM_004252334	TC226977		cytochrome p450	0.69070155	0.01700186
AK322397			atp binding	0.68421009	0.03345707
AI780377		Solyc02g072400.1.1	serine-threonine protein	0.68266362	0.02545931
AK329920			uncharacterized protein	0.6803316	0.03473793
AK326381	TC226535	Solyc09g007750.2.1	atp binding	0.67777135	0.02178015
AK321949	TC240663	Solyc03g119250.2.1	calmodulin binding protein	0.67252272	0.03651289
AI896641		Solyc11g012260.1.1	uncharacterized protein	0.66781671	0.01609183
AK323971	TC239227	Solyc08g082110.2.1	probable wrky transcription factor 41	0.66653426	0.02448464
AK329526	TC217904		auxin-induced protein	0.66601981	0.02736339
AK320098	TC228534		udp-glucose glucosyltransferase	0.66487521	0.01814823
AK324830		Solyc03g031730.2.1	beta-glucosidase 47	0.66328719	0.01531596
GO372605	TC225453		two-component response regulator arr5	0.6619064	0.01695193
AK327381	TC226022	Solyc11g066890.1.1	arogenate dehydratase prephenate dehydratase chloroplastic-like	0.65928093	0.02621399
AI486345			uncharacterized protein	0.65711382	0.02475616
BW688367	TC219408	Solyc07g005380.2.1	s-norcoclaurine synthase-like	0.65662477	0.02736339
BT012720	TC218326		glutamine synthetase	0.65239002	0.01392768
DB694012			uncharacterized protein	0.65207772	0.02385977
AW979468		Solyc03g113940.2.1	calmodulin binding protein	0.65147913	0.04915587
AK322618	TC240616	Solyc10g008410.1.1	e3 ubiquitin-protein ligase rma1h1-like	0.6502083	0.01940886
CK715255	TC223890		cinnamic acid 4-hydroxylase	0.64956376	0.03651629
AK329686	TC231955		cinnamyl alcohol dehydrogenase	0.64930446	0.03014318
BI422154			uncharacterized protein	0.64904029	0.02408902
AW038788	TC236357		uncharacterized protein	0.64867748	0.031254
XM_004246001			aspartic proteinase nepenthesin-1	0.64827685	0.01814823
ES892372	TC236456	Solyc05g055700.2.1	uncharacterized protein	0.64806584	0.01045174
EG553421	TC226835	Solyc04g078660.1.1	n-hydroxycinnamoyl benzoyltransferase	0.64791909	0.02368684
AK329749	TC242527		uncharacterized protein	0.64369381	0.01453292
BG127730			ca2+ antiporter cation exchanger	0.64054888	0.0366091
AK324054			glycosyl hydrolase-like partial	0.63965778	0.02736339
AY150039	TC232665		kda class i heat shock protein	0.6374395	0.01513849
BI206751	TC224791		cytochrome p450	0.63732582	0.01509349
AW442693			auxin-induced protein	0.63686551	0.03042268
BP889943	TC243859		uncharacterized protein	0.63443146	0.01801284
AK324002	TC219586		lignin-forming anionic peroxidase	0.63421795	0.02338954
DB714953			sulfite exporter family protein	0.630419	0.031254
BE431491		Solyc03g058950.2.1	phosphoglycerate mutase-like protein	0.63021789	0.0218833
CK715255			cinnamate 4-hydroxylase	0.63015062	0.0420314
AF211790			uncharacterized protein	0.62964436	0.04602306
BE431646			trans-cinnamate 4-hydroxylase	0.62788842	0.04260118
BI421287		Solyc03g007380.1.1	uncharacterized protein	0.62773445	0.0402595
AK319838	TC223430	Solyc07g062490.1.1	epidermis-specific secreted glycoprotein ep1	0.62538407	0.01513849
AK323113	TC232016	Solyc07g043490.1.1	udp-glucose:solanidine glucosyltransferase	0.62518881	0.01513849
AW932015			uncharacterized protein	0.62496577	0.01814823
AW443102	TC233725		uncharacterized protein	0.62455924	0.01814823

BT013355	TC223627		pathogenesis-related protein 4	0.62418564	0.04017979
AK321158	TC241857	Solyc08g068620.1.1	histidine decarboxylase	0.62388333	0.03651629
XM_004233377	TC233716		cytochrome p450	0.6224384	0.02715404
AK328428	TC229400	Solyc03g083960.2.1	haloacid dehalogenase-like hydrolase domain-containing protein	0.62099277	0.01129466
AK247332	TC220494	Solyc11g015890.1.1	f-box protein	0.61892333	0.02907994
FS187400	TC227584	Solyc10g085950.1.1	protein aluminum sensitive 3	0.61758707	0.04004804
BI924118		Solyc02g071710.2.1	GDSL esterase/lipase At1g29670	0.61549818	0.02178015
AI487784			gdsl esterase lipase	0.61423381	0.04397335
AK322690	TC241030		ring finger family protein	0.61175454	0.02140228
AK327303	TC231062		zinc finger ccch domain-containing protein	0.61164933	0.02787856
BT013052	TC235672		proline rich protein	0.61163615	0.01728035
AI488552			uncharacterized protein	0.61052646	0.031254
DB700370	TC239751	Solyc06g006110.2.1	vacuolar cation proton exchanger	0.60959379	0.04014828
XM_004246294	TC239102	Solyc09g013150.2.1	probable anion transporter chloroplastic-like	0.60879276	0.03575395
AK320809	TC237807	Solyc04g009960.2.1	threonine aldolase	0.60860693	0.02073976
AK323007	TC217660	Solyc06g082590.1.1	ap2 erf domain-containing transcription factor	0.60685429	0.02787856
AK328730	TC231134	Solyc12g100150.1.1	LOB domain protein 4	0.60664586	0.03014318
BT014571			act domain-containing protein	0.60462285	0.01940886
GT164180	TC231555		1-deoxy-d-xylulose 5-phosphate synthase	0.60391065	0.0218833
DV105517		Solyc07g063390.2.1	beta-glucosidase 18-like	0.60301157	0.0113692
BT013058	TC224390		erg28 like protein	0.60294078	0.01392768
BI421969			inosine-uridine preferring nucleoside hydrolase family protein	0.60222164	0.04344963
CK714819	TC220503		abhydrolase domain	0.59972142	0.03561419
BW688043			type-a response regulator	0.59620178	0.01463349
AK322078			glycerol-3-phosphate transporter 5-like	0.59557069	0.031254
AI485590			cucumber peeling	0.59540236	0.01513849
XM_004250354	TC229000		lignin-forming anionic peroxidase	0.59440716	0.0340683
AA824883		Solyc03g007760.2.1	uncharacterized protein	0.59379124	0.0250884
XM_004236380	TC239209		auxin-induced protein 5ng4-like	0.59172481	0.02533161
AW037462			tyrosine specific protein phosphatase family protein	0.59092209	0.01932909
AK323040	TC220781	Solyc07g055710.2.1	heat shock factor	0.58897648	0.03765038
XM_004235770		Solyc03g119520.2.1	uncharacterized protein	0.58793155	0.0136293
U66300	TC238942	Solyc03g082420.2.1	chloroplast small heat shock protein	0.58565127	0.02621399
BI422058	TC223662		arogenate prephenate dehydratase	0.58564228	0.03765038
AK322970	TC225589	Solyc11g007730.1.1	phosphorylase family protein	0.58353011	0.02292687
AK326828	TC229189		flavonoid 3-glucosyl transferase	0.58241276	0.01824748
BG631811			uncharacterized protein	0.57753273	0.01513849
AK325911	TC219980	Solyc05g016310.1.1	aspartic proteinase nepenthesin-2-like	0.57725009	0.02514321
BT014384	TC232347		flavonol synthase flavanone 3-	0.57495888	0.02500165
BT014279	TC238580		monoglyceride lipase	0.57472087	0.031254
AK247484	TC243465	Solyc01g091320.2.1	c-4 sterol methyl oxidase	0.57439748	0.03733858
AC215480	TC221384	Solyc02g093250.2.1	caffeoyl- o-methyltransferase	0.57415469	0.03968271
AI896828			uncharacterized protein	0.57386175	0.03880424
AK326432	TC234915	Solyc05g009120.2.1	uncharacterized protein	0.57382476	0.01890347
BP877587			beta-glucosidase 44-like	0.57248102	0.02715404
DB709383		Solyc10g076480.1.1	ammonium transporter	0.57126448	0.0402219
BT013655	TC242170	Solyc11g006410.1.1	uncharacterized protein	0.57098814	0.02172422
AK323846	TC239845	Solyc06g083130.2.1	MazG nucleotide pyrophosphohydrolase	0.56919275	0.0376923
AK320895	TC231443	Solyc03g025390.2.1	rossmann-fold nad -binding domain-containing protein	0.56822017	0.04951034
BG131246	TC237643		apyrase	0.56820407	0.01463349

AK323811	TC223602	Solyc08g077020.1.1	uncharacterized protein	0.56792528	0.01647345
XM_004241581	TC236549		lipid binding	0.56774914	0.03275513
AK320220	TC220490	Solyc02g092580.2.1	peroxidase 73	0.56476991	0.03880424
AK324331	TC235051	Solyc11g010100.1.1	gtp-binding protein	0.56427811	0.02002032
BG127254			uncharacterized protein	0.56374285	0.02509365
BI926166			secologanin synthase-like	0.56323543	0.04444052
AK328307			glucomannan 4-beta-mannosyltransferase 9-like	0.56060651	0.0283751
DB696023	TC228652		uncharacterized protein	0.5586268	0.02280798
AK247983	TC225553		uncharacterized protein	0.55840757	0.02338426
AK321958	TC217531	Solyc03g117250.2.1	flotillin-like protein	0.55764334	0.02509365
AK324555	TC219804	Solyc02g093230.2.1	caffeoyl- o-methyltransferase	0.55704963	0.02736339
BG131008			proline rich protein	0.55700672	0.0433385
BF114318		Solyc10g085380.1.1	sex determination protein tasselseed-2-like	0.55485467	0.02514321
AC216704			ninja-family protein afp3	0.55467457	0.0136293
AW216413	TC236372		s-adenosylmethionine synthetase	0.55448704	0.02042812
XM_004252080	TC241138		cellulose synthase-like protein e6-like	0.55412545	0.04667545
AK321165			probable leucine-rich repeat receptor-like protein kinase at1g68400-like	0.55089011	0.02966005
BG628296			uncharacterized protein	0.55044898	0.02207648
AW648982		Solyc07g045440.1.1	fasciclin-like arabinogalactan protein	0.54989944	0.03016826
AP009260	TC234617		60s ribosomal protein l13	0.54969294	0.02736339
EG552932	TC233416		uncharacterized protein	0.54893444	0.01824748
XM_004231528	TC244295	Solyc02g030300.2.1	g-type lectin s-receptor-like serine threonine-protein kinase at4g27290-like	0.54758085	0.01840576
AK322358	TC228265	Solyc02g080890.2.1	wrky transcription factor 6-like	0.54599995	0.04217752
AK327871	TC219924		brassinosteroid-regulated protein bru1	0.54580088	0.0402595
AK326022	TC243613	Solyc04g040180.2.1	methyltransferase ddb_g0268948-like	0.54509674	0.04811726
AK324897	TC224303	Solyc08g065150.1.1	uncharacterized protein	0.54369863	0.02509365
AK247331	TC222269	Solyc11g066680.1.1	zeatin o-glucosyltransferase-like	0.54242527	0.03470553
EG553495	TC228441	Solyc08g078900.1.1	protease inhibitor/seed storage/lipid transfer protein (LTP) family protein	0.54106831	0.02450945
XM_004249215	TC236687		24-sterol c-methyltransferase	0.53758297	0.0368007
AK329374	TC230806	Solyc09g009660.2.1	solute carrier family 35 member f1-like	0.53634231	0.01940886
AK322844	TC239263	Solyc01g010000.2.1	uncharacterized protein	0.53561497	0.02860306
AI775181	TC217355		ascorbate peroxidase	0.5335653	0.02736339
BI203889			uncharacterized protein	0.53332981	0.02450945
AK323625	TC226469		nadph:quinone oxidoreductase	0.5305919	0.02450945
AK326842	TC244366		type-a response regulator	0.5298755	0.03004499
BI922321			auxin-induced SAUR-like protein	0.52898281	0.01823317
AK327096	TC242691	Solyc09g061860.2.1	transferring glycosyl	0.52847901	0.04411017
BT014417	TC224988		l-ascorbate oxidase homolog	0.52617038	0.02503639
AW626285	TC234801		uncharacterized protein	0.52554478	0.031254
AK319920	TC228340	Solyc01g110290.2.1	squalene synthase	0.52385989	0.02545931
AK319948	TC228759		heparanase-like protein 3-like	0.52330313	0.04953338
AK319503	TC242239		3-isopropylmalate dehydrogenase	0.52296399	0.02627242
XM_004240990		Solyc06g053930.2.1	calmodulin-like protein	0.51833656	0.01823317
AW030968			uncharacterized protein	0.5183104	0.02178015
GO374941	TC234820		spermidine synthase	0.51825675	0.04951034
DB717666			uncharacterized protein	0.51807803	0.02521258
AA824893			uncharacterized protein	0.51661152	0.03880424
AK322919		Solyc04g005050.1.1	matrix metalloprotease domain-containing protein	0.51638603	0.04070823
AK322540	TC232963		3-beta hydroxysteroid dehydrogenase	0.51579811	0.02388603
AK247718			acyl- n-acyltransferases-like protein	0.51529105	0.01695193

AK323518	TC236796		transcription factor bhlh62-like	0.51411876	0.01700186
GH622685	TC217624	Solyc06g076050.2.1	e3 ubiquitin-protein ligase xbat31-like	0.51382705	0.02521258
BG134081		Solyc10g008000.1.1	Light-dependent short hypocotyls 1	0.51209384	0.04624782
BI422114	TC224272	Solyc09g011870.1.1	arogenate dehydrogenase chloroplastic-like	0.51195789	0.03886765
XM_004242757	TC225326		nucleotide pyrophosphatase phosphodiesterase	0.51003783	0.04090419
AK321863	TC230878		probable ribose-5-phosphate isomerase-like	0.51003486	0.031254
AK327726	TC237035		6-phosphogluconate dehydrogenase	0.50997315	0.03032666
AK328809	TC236869	Solyc06g009510.1.1	transcription factor transcription regulator	0.50968782	0.0340683
AK328708	TC221450		gtp-binding protein	0.50726526	0.031254
ES892135		Solyc10g079600.1.1	type-a response regulator	0.50654759	0.0382606
AK325882	TC228088		uncharacterized protein	0.50651621	0.0352324
AK328438	TC224533		4-coumarate: ligase	0.5061737	0.03914168
CK348391			uncharacterized protein	0.50521719	0.02521258
GT167514			uncharacterized protein	0.50408365	0.01728035
XM_004238678		Solyc05g006420.2.1	type-a response regulator	0.50336044	0.04661723
BE434445	TC230518	Solyc05g054320.2.1	epoxide hydrolase	0.50212208	0.03510861
AK319823	TC229568	Solyc12g006460.1.1	ent-kaurenoic acid oxidase 2	0.50189076	0.0218833
AK323682	TC218071	Solyc06g009020.2.1	glutathione s-transferase	0.50111642	0.04017979
X59883			glycine-rich rna binding protein	0.50097805	0.03204623
BG127662	TC231494	Solyc10g083970.1.1	s-adenosylmethionine synthetase	0.49930477	0.04444052
AK326904	TC220586		fumarylacetoacetase	0.49741734	0.031254
AW443419	TC227819		uncharacterized protein	0.4969031	0.03213262
BI211145	TC237490		xyloglucan endotransglucosylase hydrolase 7	0.49680259	0.04953338
XM_004245630	TC217204	Solyc08g079880.1.1	serine protease	0.49613365	0.04004804
BE450178	TC236494		bag-domain protein 1 regulator of cell death	0.49604119	0.03684043
AW649155			act domain-containing protein	0.49406627	0.02521258
BT013853	TC239149	Solyc05g005950.2.1	probable peptide transporter at1g52190-like	0.49401029	0.02627242
BG129166	TC235096		expansin precursor	0.49316699	0.03914168
DB714303			uncharacterized protein	0.49240329	0.02061969
AK224857	TC223918	Solyc02g088910.2.1	ninja-family protein afp3	0.49040585	0.02509365
AK325571	TC227206	Solyc07g005760.2.1	hydroxycinnamoyl quinate transferase	0.48835821	0.03192294
AK327971	TC229286		calmodulin-like protein	0.48720473	0.03942635
BE353787			uncharacterized protein	0.48663631	0.04014828
AK328919	TC234789	Solyc08g005090.1.1	uncharacterized protein	0.4852982	0.02855956
AK320178			cytochrome p450 83b1-like	0.48336539	0.03575395
XM_004242883	TC224236	Solyc07g019460.2.1	cytochrome p450 reductase	0.4820915	0.02736339
AK322577	TC245149	Solyc03g112970.1.1	uncharacterized protein	0.48127131	0.04081726
GT164027			uncharacterized protein	0.48091174	0.03968271
BT014282	NP12919594	Solyc06g007130.2.1	omega-3 fatty acid desaturase	0.48012781	0.02292687
BI921549	TC243565		glycosyltransferase family 61 protein	0.47917946	0.0218833
XM_004248080	TC240211		gtp-binding protein	0.47909173	0.031254
ES895349	TC240342	Solyc03g114460.2.1	acyl-activating enzyme 17	0.47903852	0.02736339
AK323065	TC232387	Solyc02g067440.2.1	uncharacterized protein	0.47792155	0.04802333
AK224857	TC230530		ninja-family protein afp3	0.47657346	0.02545931
AK325389	TC234090		udp-glucose dehydrogenase	0.47587241	0.03042268
AW219705		Solyc08g079550.1.1	uncharacterized protein	0.47586976	0.03780872
AK247430	TC232482	Solyc05g006780.2.1	actin cross-linking protein	0.47436635	0.0433216
AK324141	TC232420	Solyc02g063390.2.1	uncharacterized protein	0.47402316	0.03241277
AK320053	TC231722	Solyc11g066820.1.1	glucomannan 4-beta-mannosyltransferase 2	0.47401269	0.03014318
AC244298	TC237972		calcium-dependent protein	0.47368976	0.02660843

AW218373			b12d-like protein	0.4733098	0.04772778
BG134424		Solyc03g006220.2.1	protein trichome birefringence-like 38	0.47311619	0.02140228
BT013258	TC229631		dynamin-related protein 1e	0.47292123	0.02450945
AK320212	TC217409		s-adenosyl-l-methionine synthetase	0.46893319	0.03914168
AK328892	TC241957	Solyc06g050500.2.1	abscisic acid receptor pyl4	0.46865765	0.02784704
BP889010			uncharacterized protein	0.46855885	0.02178015
BT012939	TC227099	Solyc04g051860.2.1	shikimate kinase	0.46779048	0.03780872
GT166303			uncharacterized protein	0.46570382	0.03491406
AK322433	TC218959	Solyc07g056510.2.1	glutathione s-transferase	0.46546837	0.03914168
BI935704			secologanin synthase-like	0.46529466	0.03518348
AK320908	TC223300	Solyc09g083280.2.1	uncharacterized protein	0.46505788	0.02909651
BI933296			auxin-responsive protein iaa7	0.46394609	0.04915587
AK329436			pi-plc x domain-containing protein at5g67130-like	0.46278764	0.04442187
AK321308	TC229183	Solyc01g107670.2.1	leucine-rich repeat disease resistance protein	0.4617629	0.02140228
FS204922		Solyc08g063130.2.1	3-hydroxybenzoate 6-hydroxylase 1-like	0.46060789	0.03765038
DB691528	TC229671	Solyc01g110060.2.1	purple acid phosphatase	0.46033338	0.04143213
GO375240			bZIP transcription factor	0.45955676	0.0402595
AK322988	TC241252		uncharacterized protein	0.45780536	0.03592504
XM_004244225	TC238921		secologanin synthase-like	0.45775229	0.031254
BG140300			uncharacterized protein	0.45730793	0.04397335
AW624867		Solyc09g075680.1.1	2-hydroxyisoflavanone dehydratase	0.45709631	0.04921428
DB719859	TC221746	Solyc02g082550.2.1	protein kinase	0.45628379	0.03657075
DB684794			aminotransferase y4ub	0.45597935	0.04921428
BW691431			uncharacterized protein	0.45548059	0.031254
AF059489	TC219221	Solyc02g088100.2.1	expansin	0.45464563	0.0376923
BE459640			uncharacterized protein	0.45406175	0.02660843
AK247484			c-4 sterol methyl oxidase	0.4522931	0.03330195
AK326917	TC227487	Solyc08g008280.2.1	uncharacterized protein	0.44985089	0.04444052
AK327796	TC226466		vacuolar atp synthase 21 kda proteolipid subunit	0.44973575	0.03880424
AW032389	TC222470	Solyc02g080070.2.1	receptor-like serine-threonine protein kinase	0.4491839	0.03576305
AK328380	TC225665		phosphate phosphoenolpyruvate translocator	0.4491474	0.0352324
AK321464	TC232149	Solyc11g066830.1.1	u2 snrnp auxiliary small	0.44619208	0.02736339
AK329953	TC218188		rna recognition motif-containing protein	0.44599456	0.03880424
AK326646	TC222681		uncharacterized protein	0.44526649	0.02345875
AI486252			tyrosine-specific transport	0.44439089	0.02909651
DB726092	TC228835		uncharacterized protein	0.44339671	0.0420314
BT014069	TC218881	Solyc08g041770.2.1	uncharacterized protein	0.44330447	0.03968271
BW688261	TC217617	Solyc09g064940.2.1	phenazine biosynthesis	0.442307	0.02664653
AJ002590	TC224441	Solyc04g078470.2.1	d3-type cyclin	0.44152057	0.02540338
BT013379			glucose-6-phosphate 1-epimerase-like	0.44012695	0.04025063
AI780199			uncharacterized protein	0.43964352	0.03914168
DV105571	TC231325		uncharacterized protein	0.43938557	0.04624782
BI205061			l-ascorbate oxidase homolog	0.43886075	0.03839557
AW623604	TC244799		uncharacterized protein	0.43885463	0.03518348
AK324347	TC231671	Solyc11g066060.1.1	heat shock protein 70	0.4380342	0.02503639
BI204050	TC242557	Solyc03g031800.2.1	xyloglucan endotransglucosylase hydrolase protein a	0.43739969	0.04286715
AW154840	TC245259		uncharacterized protein	0.43717627	0.04802333
AJ831477			uncharacterized protein	0.43714538	0.04017979
BW686429	TC218485	Solyc02g087770.2.1	aldose 1-epimerase	0.43675917	0.02636752
AK326383			uncharacterized protein	0.43636843	0.03886765

AI637314			uncharacterized protein	0.43611576	0.03588922
AK326688	TC230885		monocopper oxidase-like protein sku5	0.43560749	0.03733858
AK323203	TC217324		mitogen-activated protein kinase kinase 5	0.4354003	0.0340683
XM_004237845	TC222512		endoplasmic reticulum vesicle transporter protein	0.43489434	0.04017979
XM_004228962	TC231758	Solyc01g059880.2.1	atp-citrate synthase beta chain protein 1-like	0.43474202	0.03503395
XM_004236986		Solyc04g005670.1.1	kelch repeat-containing f-box family protein	0.4336903	0.04017979
GT163523			uncharacterized protein	0.43343569	0.04802333
AK320967	TC244827		methylenetetrahydrofolate reductase	0.43338787	0.03880424
AK319588	TC218321		isocitrate dehydrogenase	0.4332856	0.02621399
BI210488	TC233207		phosphoglucomutase	0.43314245	0.02715404
AK327370	TC225945	Solyc08g075230.1.1	Genomic DNA chromosome 5 P1 clone MDA7	0.43313757	0.03213262
AI895966			phenazine biosynthesis	0.43269343	0.02545931
XM_004253452		Solyc00g206460.1.1	uncharacterized protein	0.43197401	0.04953338
AK326427	TC234206		protein tify 6b-like	0.43188601	0.03677927
BG735249			uncharacterized protein	0.43178793	0.03051034
AK327187	TC223658	Solyc12g013690.1.1	fad nad -binding oxidoreductase family protein	0.42878431	0.03462098
AK323740	TC217246		1-aminocyclopropane-1-carboxylate synthase	0.42856214	0.04213426
AW621890	TC232400		acetyl- cytosolic	0.42804782	0.04014828
AK324936			mevalonate kinase	0.42569143	0.04046689
EF151131	TC222707		uncharacterized protein	0.42522225	0.03968076
BP908190			uncharacterized protein	0.4248563	0.03880424
AK323579	TC234071		probable 3-beta-hydroxysteroid-delta -isomerase-like	0.42409389	0.031254
BP890506			uncharacterized protein	0.42361164	0.02540338
AK325637	TC217699		copper ion binding	0.42267593	0.03958396
AK324581	TC237549	Solyc11g010500.1.1	mitochondrial dicarboxylate carrier	0.42239095	0.03474152
BT012975	TC228133		annexin p35	0.42188829	0.03334823
BT013237	TC235420		beta-d-glucan exohydrolase-like protein	0.42174252	0.0376923
AW944799			pyruvate cytosolic isozyme-like	0.42144447	0.03588922
AC233133	TC243063		aminotransferase class IV family protein	0.42017387	0.03576305
EG553638	TC244519		uncharacterized protein	0.41989386	0.04735608
BI935660		Solyc10g084930.1.1	protein kinase	0.41975399	0.03518348
AK326055	TC235019		methyltransferase pmt9	0.41768873	0.04130896
AK323013	TC240689	Solyc03g116710.2.1	GTP binding protein	0.41737399	0.0420314
AI485975	TC229216		beta- -xylosidase	0.41734428	0.04209266
XM_004239985			endo- -beta-xylanase c	0.41696006	0.03880424
AW037866			uncharacterized protein	0.41634116	0.02907994
AI484010	TC228626		bzip transcription factor	0.41624788	0.04792261
GO372328	TC231321		glutaredoxin	0.41517619	0.04269999
AK328036	TC233970		zinc finger protein	0.41472861	0.04921428
DB678717	TC224802		embryo-specific protein	0.4142639	0.04017979
AK247712			dehydroquinate dehydratase shikimate dehydrogenase	0.41410667	0.04070823
AW219445	TC240036	Solyc01g073880.2.1	rrna intron-encoded homing endonuclease	0.41390552	0.03510861
ES894904	TC225585		uncharacterized protein	0.41295323	0.0360622
DV104791			uncharacterized protein	0.41255612	0.04336363
AK323190	TC232806	Solyc03g117330.2.1	uncharacterized protein	0.41148933	0.02707424
AC216910	TC230766		alcohol dehydrogenase class iii	0.41045635	0.04070823
AK322250	TC231972		aromatic and neutral amino acid transporter	0.40752518	0.02890416
AK323973			uncharacterized protein	0.40615656	0.03968271
AW033973	TC237370		6-phosphofructokinase chloroplastic-like	0.40498494	0.031254
AK319852	TC232840	Solyc01g100000.2.1	MEE66 (maternal effect embryo arrest 66)	0.40478679	0.0352324

AK323168	TC235026		20s proteasome subunit paf1	0.40371863	0.03749231
AK326131	TC235679		xyloglucan galactosyltransferase	0.40321105	0.04661723
AW032973			u-box domain-containing protein 4-like	0.40137954	0.04191195
AW219022	TC230272		mads-box protein	0.40129173	0.02798648
BI921863	TC235246	Solyc02g067800.2.1	lob domain-containing protein 25	0.40115927	0.04143213
BI422137	TC227221	Solyc09g014990.2.1	wrky-type transcription factor	0.40073805	0.03880424
AK323990	TC223096		type-a response regulator	0.39924691	0.04070823
AK320306	TC235143		membrane protein	0.39912123	0.04758869
AK320450	TC237031	Solyc04g070980.2.1	cycloartenol synthase	0.39759125	0.03491406
AC217002	TC223619		udp-d-apiose udp-d-xylose synthase 1	0.39755346	0.03859621
AK320874	TC221959	Solyc12g045020.1.1	cytochrome p450	0.3973908	0.0399106
AF124139	TC218004		uncharacterized protein	0.39731143	0.031254
AI898979			70 kda peptidyl-prolyl isomerase	0.3956785	0.03505235
BP904822			uncharacterized protein	0.3953925	0.0397115
AK325190	TC239316		udp-galactose transporter 2-like	0.39506202	0.03241277
BG631767			uncharacterized protein	0.39165823	0.03470553
BI423148	TC236084	Solyc08g014570.2.1	ankyrin repeat-containing protein at5g02620-like	0.39101062	0.03968076
AK322703	TC220192	Solyc03g112540.2.1	rossmann-fold nad -binding domain-containing protein	0.39076532	0.031254
AW219448		Solyc05g018110.1.1; Soly	Ulp1 protease family C-terminal catalytic domain containing protein	0.39005085	0.03241277
GT165053	TC229420		probable peptide transporter at1g52190-like	0.38760582	0.03576305
AK320937	TC228726		acetyl- c-acetyltransferase	0.38706904	0.03474152
AW625543			uncharacterized protein	0.38378445	0.03886765
AK327640			uncharacterized protein	0.38377295	0.04151738
DV105061			uncharacterized protein	0.38373864	0.04624782
AW649514	TC244934		methionine synthase	0.38323239	0.04958531
XM_006342039	TC230437	Solyc04g082130.2.1	trna pseudouridine synthase pus10-like	0.38316402	0.04004804
AK324127	TC226646		protein notum homolog	0.38312581	0.04297324
AK328499	TC237652		glucan endo- -beta-glucosidase 4-like	0.38301449	0.04830117
BI933507	TC240895		atp-citrate lyase a-1	0.37985123	0.04245491
AK247870	TC226773		s-type anion channel slah3-like	0.37982199	0.03820904
AK325212	TC217440	Solyc06g034110.2.1	acid phosphatase	0.37970215	0.04070823
U21800	TC239214		hyoscyamine 6 beta-hydroxylase	0.37968272	0.0420314
AJ784637	TC242194		uncharacterized protein	0.37951705	0.04360618
AK323335	TC225624		cytochrome c	0.37946425	0.0382606
AK320360	TC219384	Solyc10g083940.1.1	major facilitator protein	0.37800065	0.04531512
AK319596	TC235856		adenylosuccinate synthetase	0.37636347	0.04311359
AK322827	TC235383	Solyc12g008650.1.1	myo-inositol oxygenase	0.37532534	0.03880424
BI926404		Solyc01g107180.2.1	btb poz domain-containing protein npy2-like	0.37453619	0.03791821
AJ277944	TC228062		uncharacterized protein	0.3732642	0.04164235
XM_004232986	TC241771		u-box domain-containing protein 17-like	0.37071087	0.03886765
AI895309		Solyc03g006080.2.1	serine-threonine protein plant-	0.37045909	0.04130896
AK324941	TC217783		farnesyl diphosphate synthase	0.37008026	0.03942635
AK325476			u3 small nucleolar rna-associated protein 18 homolog	0.3687848	0.03880424
AK322292	TC238277		snare associated golgi protein	0.36863135	0.04424732
BF096327		Solyc03g120660.2.1	hydroxyphenylpyruvate reductase	0.36835181	0.04117608
AK329598	TC237358	Solyc00g050430.2.1	transcription factor bhlh93	0.36696075	0.04070823
DB708167	TC224886	Solyc01g091130.2.1	nitroreductase-like protein	0.36694506	0.03732846
AW036165			uncharacterized protein	0.36671666	0.04188161
BP911113			uncharacterized protein	0.36655138	0.04288534
DB710831	TC234803	Solyc06g075610.1.1	protein binding	0.36567895	0.04311359

AK327809	TC221542		uncharacterized protein	0.36558231	0.04530457
AK321560	TC228819	Solyc12g015880.1.1	heat shock protein 90	0.36408823	0.04399938
BT012792	TC240006		calcium-dependent protein kinase	0.36370949	0.04651407
GO373561	TC235403		nadh-cytochrome b5 reductase	0.36365896	0.0382606
AK322729	TC235564		beta-ig-h3 domain-containing protein	0.36258189	0.04915587
ES896542	TC238553		caax prenyl protease	0.3614833	0.03995041
BG628653			uncharacterized protein	0.36144475	0.0488478
DB690492			erg28 like protein	0.36063479	0.04667545
CD002712	TC233413		uncharacterized protein	0.36006525	0.04897402
BI208606			14-3-3-like protein	0.35865048	0.04217752
AC246677	TC226076		nadh dehydrogenase	0.3582599	0.03893604
GT165145	TC219365	Solyc01g067000.2.1	uncharacterized protein	0.35721003	0.04772663
GO376270	TC226698		14-3-3 protein	0.35711762	0.03957817
AK325550			beta-ketoacyl-coa synthase family protein	0.35700482	0.04085008
AW624068	TC232368		hyoscyamine 6 beta-hydroxylase	0.35590993	0.04709
AK247401	TC233365		gata transcription factor	0.35543395	0.04451332
AW217373			uncharacterized protein	0.35476516	0.03886765
DB710690			gtp-binding protein	0.35460417	0.03657075
ES895259	TC228558		cytochrome b5	0.35385485	0.04709
AK323257	TC219651	Solyc01g109720.2.1	uncharacterized protein	0.35262805	0.04844452
BT014295			glutathione reductase	0.35032716	0.04164597
BI930044			uncharacterized protein	0.35023982	0.03968076
DB713872			uncharacterized protein	0.34979507	0.03714318
XM_004238973		Solyc05g011920.2.1	act domain-containing protein	0.34889618	0.04984299
AK328310	TC226620	Solyc03g062830.2.1	nudix hydrolase chloroplastic-like	0.34889563	0.04297324
AK326761	TC229776	Solyc06g071540.2.1	uncharacterized protein	0.34214855	0.0434884
AK322417	TC223808		thioredoxin h2	0.34196009	0.04453379
AW038260			uncharacterized protein	0.34067382	0.04824376
BT014563	TC226145		uncharacterized protein	0.34016739	0.04081286
AK247615			sphingoid base hydroxylase 2	0.3398818	0.0434178
BE458334	TC235649	Solyc08g079530.2.1	cpd photolyase	0.33124656	0.0433216
AY013255	TC218059		phospholipase d	0.33123326	0.04542311
AK319688	TC235472		peroxidase 5-like	0.32916466	0.04826918
AK247006	TC232895	Solyc01g087890.2.1	protein pns1	0.3241649	0.04429305
AK323460	TC223756		gamma carbonic anhydrase like 1	0.32306052	0.04522901
XM_004246391		Solyc09g010970.2.1	beta carbonic anhydrase 5	0.3194881	0.04830117
BT012699	TC237876		s-adenosyl-l-methionine synthetase	0.31523807	0.04844452
BI926080			uncharacterized protein	0.31205933	0.04830283

Supplementary Table S2. Differentially down-regulated expressed genes in the *vegetative inflorescence* (*mc-vin)* mutant relative to the wild-type Moneymaker cultivar (adjusted p-value <0.05)

GenBank Accession	TIGR Tomato Gene Index	SGN gene model ID	Gene Description	LogFC	adjusted p-value
AY306154	TC218844	Solyc05g056620.1.1	mads-box transcription factor	-4.6706063	2.35E-05
AW625684	TC239537	Solyc06g007180.2.1	asparagine synthetase	-3.07577859	0.01129466
AI491143			peroxidase-like partial	-2.14104105	0.02715404
BE460111		Solyc02g078150.2.1	plant-specific domain TIGR01615 family protein	-1.79653007	0.00494161
DV105097	TC234897		uncharacterized protein	-1.48738489	0.00107071
BG126346	TC237695		uncharacterized protein	-1.38138377	0.00550285
AK324738	TC237918	Solyc06g050320.2.1	hmg-box (high mobility group) dna-binding family protein	-1.30877409	0.02140228
BI209569			uncharacterized protein	-1.30323543	0.00132201
AW039692			DNA binding protein	-1.29480846	0.0340683
BI932387		Solyc02g079810.1.1	transcription factor dysfunctional tapetum 1-like	-1.2701243	0.00781667
BW692560	TC233191	Solyc06g068500.2.1	heat shock protein binding protein	-1.25472589	0.00529177
AK324162	TC237676	Solyc01g099990.2.1	f-box protein pp2-b15	-1.22427831	0.04792261
AJ289776	TC240464		kunitz-type protease inhibitor precursor	-1.21220033	0.02627242
AY294330	TC217461	Solyc05g015750.2.1	transcription factor	-1.20820838	0.04399253
AW221811		Solyc10g076740.1.1	isoamyl acetate-hydrolyzing	-1.20641687	0.00616242
AW220104	TC229018		uncharacterized protein	-1.19565511	0.02533161
AK247425	TC234006		fructose-bisphosphate aldolase	-1.1729753	0.02059367
AK247631	TC228498		uncharacterized protein	-1.16896361	0.01728035
AK321955	TC229919		gdsl esterase lipase cprd49	-1.16448753	0.0118309
BI203929	TC235918		uncharacterized protein	-1.11879691	0.00234593
BI203547			extensin	-1.10448619	0.00251781
AK325711	TC235595	Solyc05g012020.2.1	mads-box transcription factor	-1.06001415	0.00784769
AK247741	TC222894		uncharacterized protein	-1.05233072	0.00234593
AK325110	TC219509		fructose-bisphosphatase	-1.05132077	0.00757321
Z46675	NP000341		extensin	-1.04008001	0.00550285
AK246316	TC218602		senescence-associated protein	-1.03926825	0.00848587
ES897351	TC239970	Solyc01g088430.2.1	protein wax2-like	-1.03471875	0.00529177
AY098735	TC221404		tagl1 transcription factor	-1.02236336	0.03880424
GO372423	TC222434		auxin repressed/dormancy associated protein	-1.02151641	0.01688604
BP899244			chlorophyll a b binding protein	-1.01445743	0.02172422
DB717848	TC230994		uncharacterized protein	-1.00887786	0.0113692
BE354727			kunitz-type protease inhibitor precursor	-0.99631249	0.03491406
BP905539	TC234669		glycine-rich protein LeGRP1	-0.98568513	0.01728035
DB714089	TC219675		uncharacterized protein	-0.97575395	0.01121716
AJ459816	TC225596	Solyc06g062540.2.1	phosphoethanolamine phosphocholine	-0.96184686	0.00529177
AK321639	TC239721		uncharacterized protein	-0.95193208	0.0330068
AK247885	TC227018		ribose-phosphate pyrophosphokinase 2	-0.94697611	0.00550285
AJ538329	NP597560		protein wuschel	-0.94539119	0.03880424
AK246535	TC224738		chlorophyll a b binding protein	-0.93711381	0.02736339
BI931727	TC230794		mitogen-activated protein kinase kinase kinase 5	-0.93298563	0.01216619
BT014016			cysteine synthase	-0.93115475	0.02795052
XM_004229647	TC223079		protein wax2-like	-0.92807982	0.01129466

AK322182	TC220790	Solyc09g092260.2.1	uncharacterized protein	-0.92611605	0.02122653
BI208387	TC224432		uncharacterized protein	-0.9250578	0.02966005
AK324061	TC221778		glycine-rich rna binding protein	-0.91347756	0.01578147
X60760	NP000351		mads-box protein	-0.90652016	0.01392768
NM_001247451	TC228102	Solyc03g114840.2.1	mads-box protein 1	-0.89572786	0.0382606
BM412710	TC227173		floral-binding protein partial	-0.89543912	0.0342494
AK326775	TC232068	Solyc02g086970.2.1	aldehyde dehydrogenase family 2 member mitochondrial-like	-0.88852836	0.01513849
BG135747			uncharacterized protein	-0.88468486	0.02660843
DB708955			ml domain protein	-0.87709475	0.02855956
XM_004241153	TC232880		kda class i heat shock protein	-0.87540881	0.02475616
GO374372	TC231262		stem-specific protein tsjt1-like	-0.86635155	0.00784769
XM_004245975	TC243608	Solyc08g075150.2.1	uncharacterized protein	-0.84961806	0.0420314
AK320618	TC221287		ccch-type zinc finger protein	-0.84365961	0.03334823
AK326818	TC242871	Solyc02g094400.2.1	glycerophosphoryl diester phosphodiesterase family protein	-0.84330801	0.01840576
GO376371	TC240208	Solyc12g014630.1.1	14 kda proline-rich protein	-0.84039112	0.02450945
ES893049	TC242246		uncharacterized protein	-0.83959484	0.02073976
AK320484	TC225238		h-protein	-0.83957151	0.01840576
BG128483	TC244391		chlorophyll a b binding protein	-0.83816533	0.00550285
AW648560			uncharacterized protein	-0.82842791	0.00784769
XM_004244821	TC224495		pr-10 type pathogenesis-related protein	-0.8267964	0.02140228
DB683275	TC230723		light-regulated protein-like	-0.82572821	0.02503639
AK247674	TC219691	Solyc10g080610.1.1	f-box family protein	-0.82464748	0.04017979
AW931821			non-specific lipid transfer protein	-0.82331783	0.03345707
AC215471			aldehyde dehydrogenase family 2 member mitochondrial-like	-0.82194723	0.02338426
AK319871	TC219678		Drm3-like protein	-0.81680886	0.01184266
AW031075			chaperone protein dnaj	-0.80847264	0.00550285
DV105262			uncharacterized protein	-0.78866265	0.01628872
DV105087	TC242349		glycerophosphodiester phosphodiesterase 2	-0.78129672	0.02745311
NM_001247704		Solyc08g005680.2.1	undecaprenyl diphosphate synthase	-0.77955325	0.0263791
AW930143			protein argonaute 4	-0.77839126	0.02636752
AK247797	TC226043		uncharacterized protein	-0.77425275	0.02245621
FS203501	TC228270		uncharacterized protein	-0.76485122	0.02415464
DV105561	TC228136		pyridoxal phosphate phosphatase phospho2	-0.76281062	0.03089331
GO374372	TC217880	Solyc03g006490.2.1	stem-specific protein tsjt1-like	-0.76003022	0.01129466
AK323638	TC226071	Solyc05g012110.2.1	probable 6-phosphogluconolactonase 2-like	-0.75983439	0.01175905
AK319618	TC231891		protochlorophyllide reductase b	-0.74631066	0.03911017
BI932339		Solyc02g089170.2.1	alpha- -glucan-protein synthase	-0.74552672	0.02891439
XM_004238644	TC224123		mlp-like protein 423	-0.74546133	0.02368684
AK320619	TC236205		zinc finger protein	-0.73966661	0.03021607
AK319579	TC218921	Solyc06g069730.2.1	chlorophyll a-b binding protein 4 precursor homolog	-0.73634505	0.04602544
BE436113	TC220147	Solyc08g066240.2.1	histidine decarboxylase	-0.73165566	0.01175905
FJ797956	TC236968		dehydrodolichyl diphosphate synthase 2-like	-0.72818774	0.03632042
GO374675	TC238100	Solyc08g076730.2.1	tpr domain protein	-0.72491426	0.02552193
AI773189	TC244923		uncharacterized protein	-0.72065237	0.03732846
AK324773	TC233088	Solyc06g007580.1.1	plant-specific domain TIGR01615 family protein	-0.71870523	0.01045174
DB688269	TC219692		sigma factor sigb regulation protein rsbq	-0.71668484	0.02073976
AK247258			uncharacterized protein	-0.709711	0.0218833
XR_182712	TC226213		flavonol sulfotransferase-like protein	-0.70953923	0.031254
BT013594		Solyc08g078530.2.1	agenet domain containing protein expressed	-0.70036141	0.04714066

GT167409	TC217498	Solyc03g114560.2.1	strictosidine synthase 1-like	-0.6981436	0.01513849
EG553672	TC231056		protease inhibitor seed storage ltp family protein	-0.69737955	0.01513849
GT168321	TC230009	Solyc03g025680.2.1	PAR-1c protein	-0.69611139	0.04445268
GT167182			uncharacterized protein	-0.69540847	0.04726932
AA824781		Solyc07g054280.1.1	tryptophan decarboxylase	-0.69516893	0.02709068
XM_004250012	TC237906		vacuolar amino acid transporter 1-like	-0.69349794	0.02540338
AK325062			uncharacterized protein	-0.69149893	0.04336363
L19762	TC217174	Solyc12g009300.1.1	sucrose synthase	-0.68672895	0.02514321
AK326479		Solyc08g016310.2.1	uncharacterized protein	-0.68591836	0.04714066
AK320566	TC228209		basic 7s globulin 2 precursor small	-0.68176722	0.02715404
CD002932			peptidase m50 family	-0.67818289	0.01509349
AK323217	TC220952	Solyc12g094380.1.1	thioredoxin superfamily protein	-0.67795726	0.03390315
DB718513	TC237298	Solyc12g094640.1.1	glyceraldehyde-3-phosphate dehydrogenase	-0.67695664	0.0382606
XM_006361614	TC243575	Solyc11g008440.1.1	amino acid transporter	-0.6766177	0.02844829
AI776560			ribulose bisphosphate carboxylase	-0.67590948	0.0136293
BG791256			metallothionein-like protein	-0.66963998	0.01971002
AK320213	TC231739		uncharacterized protein	-0.66630073	0.02533161
AK325804			uncharacterized protein	-0.66541658	0.02073976
BP902454	TC241679		pectin methylesterase	-0.66519992	0.03684043
AK247861	TC218422	Solyc02g085510.1.1	Ovate protein	-0.66484071	0.03820845
AK247251	TC236012	Solyc01g109800.2.1	uncharacterized protein	-0.66480944	0.01823317
AK319577	TC217818	Solyc02g063150.2.1	ribulose bisphosphate carboxylase	-0.65694611	0.01840576
XM_004235374	TC240604	Solyc03g093370.1.1	DYAD-like	-0.65531429	0.02019611
FS189884	TC229634	Solyc07g049560.2.1	tyrosine specific protein phosphatase family protein	-0.6534043	0.01216619
BG630566	TC242392	Solyc10g080870.2.1	cytochrome p450	-0.65299152	0.04070823
AW738025			sister-chromatide cohesion protein	-0.65275037	0.01940886
AW933641			probable 6-phosphogluconolactonase 2-like	-0.65231585	0.02509365
AK323048	TC233179		metallothionein-like protein	-0.6488064	0.02237081
BE434729			uncharacterized protein	-0.64360743	0.02521258
ES895666	TC226695		thaumatin-like protein	-0.64340687	0.02736339
BG125195			putative photosystem I subunit III precursor	-0.64198147	0.0340683
AK325755	TC233034		ring u-box domain-containing protein	-0.64187242	0.02178015
BI924449			uncharacterized protein	-0.64116568	0.0391843
AK327862	TC238105		e3 ubiquitin-protein ligase chip	-0.63871554	0.0136293
BW687249			pgr5-like protein 1a	-0.63814253	0.02368684
AK323938	TC223028		uncharacterized protein	-0.63729745	0.0220667
EU057688	TC218125		histone-lysine n-methyltransferase eza1	-0.63578897	0.0218833
AW933454			uncharacterized protein	-0.62888248	0.04070823
AK322536	TC237868	Solyc06g007160.2.1	probable nadh dehydrogenase-like	-0.62731911	0.01509349
AK327947	TC217501		maternal effect embryo arrest 14 protein	-0.62608114	0.02540922
AW650793			psi reaction center subunit iii	-0.62563438	0.0189246
BP881103	TC241258	Solyc06g084140.2.1	sulfate bicarbonate oxalate exchanger and transporter sat-1	-0.62367779	0.02521258
XM_004243286	TC241603		tryptophan decarboxylase	-0.62364146	0.02545931
AK323703	TC224784	Solyc10g078920.1.1	thioredoxin-like protein	-0.62342946	0.02450945
BG630458			uncharacterized protein	-0.61802041	0.02073976
BT014213	TC236370		homeobox-leucine zipper protein hat5	-0.6163161	0.01671469
AK247326	TC228645		high mobility group family	-0.61592438	0.02475616
BG642845			uncharacterized protein	-0.61268978	0.02707424
AK321215	TC231425	Solyc03g119200.2.1	l-ala-d l-glu epimerase-like	-0.60864449	0.02338426

AK322333	TC235966		14 kda proline-rich protein	-0.60810388	0.01513849
XM_004231556		Solyc02g032300.2.1	enhancer of polycomb-like protein	-0.60781971	0.02172422
AK323321			uncharacterized protein	-0.60655025	0.02909651
AC209587	TC226824		glyceraldehyde-3-phosphate dehydrogenase	-0.60603511	0.04017979
GT168050	TC229344		uncharacterized protein	-0.60569089	0.0360622
AW649285		Solyc08g008020.1.1	uncharacterized protein	-0.60444565	0.04330572
BE432867			uncharacterized protein	-0.6014155	0.03732846
AK326785	TC223324	Solyc10g007100.2.1	mate efflux domain-containing protein	-0.60080932	0.02540338
Y08804	TC231686	Solyc00g174340.1.1	pathogenesis-related protein 1	-0.5998738	0.04271415
AK322366	TC218496		osmotin-like protein	-0.59897867	0.03914168
XM_004244391	TC236275	Solyc08g005710.2.1	ent-copalyl diphosphate synthase	-0.59833464	0.04194797
ES896200			non-specific lipid transfer protein	-0.5982845	0.031254
BI933908			light harvesting chlorophyll a b-binding protein	-0.59526647	0.02292687
AK320599	TC219213	Solyc06g064500.2.1	8-hydroxyquercetin 8-o-methyltransferase	-0.59500927	0.03859621
AK324858			probable inactive purple acid phosphatase 28-like	-0.59105949	0.02358166
AK319237	TC234470	Solyc02g085760.2.1	rhomboid-related intramembrane serine protease-like protein	-0.58856204	0.02836822
BM409036	TC222690		uncharacterized protein	-0.58668273	0.04714066
AK329542	TC229907	Solyc06g059990.2.1	dehydrodolichyl diphosphate synthase 2-like	-0.58505549	0.01824748
AK329108	TC232759		uncharacterized protein	-0.58487905	0.01823317
X98929	TC217182		subtilisin-like protease	-0.58458586	0.01761409
AW442352	TC240221		uncharacterized protein	-0.58457825	0.04595807
BT014054	TC243153		uncharacterized protein	-0.58266795	0.02049214
BI926658		Solyc03g058350.2.1	translation initiation factor eif-5b	-0.58138511	0.02178015
AK324033	TC243758	Solyc01g111170.2.1	cyclophilin	-0.57761124	0.02547194
AK326763	TC243721	Solyc09g065240.2.1	at3g63200-like partial	-0.57710222	0.01676436
DB696245			uncharacterized protein	-0.57688052	0.02521258
AK322534	TC230803		j-domain protein required for chloroplast accumulation response 1	-0.57132875	0.01513849
BI925751	TC217744		uncharacterized protein	-0.56915376	0.031254
AK321350	TC222788		uncharacterized protein	-0.56847202	0.03120964
AK322949	TC218026		dna binding	-0.56719657	0.01753217
AK320621			transmembrane fragile-x-f-associated protein	-0.56662957	0.01923585
AW094172			ribulose bisphosphate carboxylase	-0.56628614	0.03300203
BG124712	TC229817		psbP-like protein 2	-0.56507194	0.02556847
AK247469			uncharacterized protein	-0.56316906	0.02415464
XM_006348946	TC234799		inner membrane protein	-0.56309715	0.014173
BG129743	TC224883		low affinity sulfate transporter 3-like	-0.56248652	0.03886765
DB723516			glycerophosphodiester phosphodiesterase gde1-like isoform 1	-0.56173156	0.04070823
AK247342	TC218443		nuclear transcription factor y subunit b-3	-0.56022621	0.031254
BT012685	TC222146		glyceraldehyde-3-phosphate dehydrogenase a	-0.55676497	0.02897103
ES896623	TC221302		glycine-rich protein	-0.55452244	0.04406544
XM_004232308	TC245080		ribulose bisphosphate carboxylase	-0.55314255	0.03968271
BG135910			light harvesting chlorophyll a b-binding protein	-0.55048557	0.01728035
AK322999	TC219426		class i chitinase	-0.548703	0.03483294
AK247039	TC227682		uncharacterized protein	-0.54857922	0.02770329
ES895497	TC235884	Solyc02g085950.2.1	ribulose -bisphosphate carboxylase oxygenase	-0.54825507	0.0402595
ES894498			non-specific lipid transfer protein	-0.54806572	0.03204623
XM_004230320			uncharacterized protein	-0.54620389	0.0198878
XM_006365512	TC241071	Solyc02g071000.1.1	chlorophyll a b binding protein	-0.54342691	0.02597909
BI204682	TC229657		uncharacterized protein	-0.5429087	0.03740699

ES893043	TC236646		auxin response factor 2	-0.54246689	0.0340683
AW442671			ribulose bisphosphate carboxylase	-0.53748381	0.04978458
AI773231			phosphoribulokinase precursor	-0.53735255	0.02787856
ES895760	TC220114	Solyc02g084390.2.1	atp binding microtubule motor family protein	-0.5346411	0.01573238
GO375686	TC223298		non-specific lipid transfer protein	-0.53425582	0.04245491
AK324990			chloroplast-targeted copper	-0.53204525	0.0402595
BG133460			uncharacterized protein	-0.52880333	0.04645831
AI782802	TC238819		ribulose bisphosphate carboxylase	-0.52840994	0.03820845
AI775649			ribulose bisphosphate carboxylase	-0.52793994	0.02521258
AW648559			uncharacterized protein	-0.52792028	0.02660843
AW930552			uncharacterized protein	-0.52711708	0.02521258
AI774033			ribulose -bisphosphate carboxylase oxygenase	-0.52505314	0.04117608
AW034476			uncharacterized protein	-0.52449334	0.04245491
BG124604			uncharacterized protein	-0.52338005	0.03390315
XM_004243578		Solyc07g043660.2.1	Acyl-CoA synthetase/AMP-acid ligase II	-0.52044442	0.04070268
AK326729		Solyc01g111180.2.1	NHL repeat-containing protein 2-like	-0.51898346	0.02207648
AK324086	TC237151		gibberellin induced protein	-0.51785006	0.04542311
XM_004244682	TC224271	Solyc08g013670.2.1	photosystem i reaction center subunit n	-0.51645749	0.04143213
BG128317	TC238479		uncharacterized protein	-0.5153303	0.02086518
BW685530			uncharacterized protein	-0.51442018	0.02892259
XM_004251752			ketol-acid reductoisomerase	-0.51431072	0.04017979
BT013590			gdp-mannose 3 -epimerase	-0.51373675	0.02767607
AK319286	TC231220	Solyc01g100540.2.1	PAPA-1-like conserved region family protein expressed	-0.51367541	0.03651629
FJ190668		Solyc02g081670.1.1	double top	-0.5113397	0.03914168
XM_004241829		Solyc06g063370.2.1	chlorophyll a-b binding protein chloroplastic-like	-0.50940405	0.03081024
BT012706	TC229661		protein iq-domain 14	-0.5090342	0.02140228
BG643539			uncharacterized protein	-0.50801725	0.02909651
GT168222			udp-glucuronate 5-	-0.50654901	0.03016237
BW691109			dynamin-related protein 3a	-0.50516776	0.03576305
XM_004245759	TC235066		main allergen 15 kda oleosin	-0.50513879	0.04014828
BP900647			chlorophyll a b binding	-0.50460944	0.02475616
AK321362	TC228761	Solyc12g008980.1.1	lycopene epsilon cyclase	-0.50427754	0.02475616
BE460487	TC238244		uncharacterized protein	-0.50220588	0.04661723
BW686795			acyl- -binding domain 3	-0.50013427	0.031254
AC215381	TC227186	Solyc02g070950.1.1	light harvesting chlorophyll a b-binding protein	-0.49895571	0.02368684
AJ850958	TC217175		alpha dioxygenase	-0.49743121	0.04424732
AW622830			uncharacterized protein	-0.49609528	0.04624782
CD002727	TC231267		uncharacterized protein	-0.49094205	0.02521258
XM_004240326		Solyc06g005710.2.1	protein plastid transcriptionally active 16	-0.49087832	0.0352324
AI899395			uncharacterized protein	-0.49074583	0.03042268
ES896520			non-specific lipid transfer protein	-0.49007618	0.04596287
AW038831			uncharacterized protein	-0.48997616	0.031254
DB716492	TC229504		fzo-like protein	-0.48916204	0.04272739
AK321256	TC229663		uncharacterized protein	-0.48888008	0.03338942
BT013775			aspartic proteinase	-0.48833426	0.02736339
BT014515	TC219361	Solyc09g009940.2.1	signal recognition particle 54 kda chloroplastic-like	-0.48689635	0.02621399
BE344485			uncharacterized protein	-0.48671841	0.04143213
AW223874	TC241055	Solyc12g070080.1.1	glutamyl-trna amidotransferase subunit a-like	-0.48654278	0.03911017
DB723972			60s ribosomal protein l27a	-0.48597339	0.02521258

GO374193	TC232512	Solyc10g006230.2.1	light-harvesting complex i protein lhca2	-0.48585774	0.02408902
AW617563			uncharacterized protein	-0.48548507	0.02627242
AK329555	TC231464	Solyc08g061260.2.1	g protein-coupled receptor	-0.48128895	0.02545931
BI929256	TC223043		ribulose bisphosphate carboxylase	-0.48083997	0.0218833
XM_004242943			uncharacterized protein	-0.48016746	0.02514321
AK329878	TC227773		uncharacterized protein	-0.48006563	0.02816051
XM_004232809	TC234451		ring finger protein	-0.47872292	0.04085008
AK328833	TC218515	Solyc05g014710.2.1	remorin family protein	-0.47839845	0.03942635
AF465780	TC221750		uncharacterized protein	-0.47803464	0.03170918
AK322173	TC220437	Solyc03g117890.2.1	amino acid binding	-0.47793674	0.03632042
XM_004245853	TC234297	Solyc08g076850.2.1	tetraspanin family protein	-0.47760207	0.03625368
AK319691	TC228859	Solyc01g100910.2.1	auxin-induced protein 5ng4-like	-0.47653025	0.02514321
AK325562			isoaspartyl peptidase l-asparaginase 1 subunit beta	-0.47610199	0.03125374
AK327849			f-box wd-40 repeat-containing protein	-0.47538035	0.0218833
X98865	TC217870		14-3-3 protein	-0.47469424	0.02871289
AW036071			uncharacterized protein	-0.47448931	0.02844829
BG132416			uncharacterized protein	-0.47340439	0.03820904
BG128157			light-harvesting complex ii protein lhcb3	-0.47262916	0.03724721
AK322603	TC226421	Solyc01g091700.2.1	f-box family protein	-0.47243098	0.01923255
EG553725	TC217649		ring-h2 finger protein atl80	-0.47048023	0.03004499
AK323436	TC224078	Solyc11g040040.1.1	phototropic-responsive nph3 family protein	-0.4697628	0.03345707
BP906823			ribulose bisphosphate carboxylase	-0.46901351	0.031254
BG130653			uncharacterized protein	-0.46846788	0.02652399
DB717672	TC237946		uncharacterized protein	-0.46830066	0.04200443
AK326493			3-hydroxybenzoate 6-hydroxylase	-0.4678975	0.02542915
AK319516	TC220146	Solyc09g011810.2.1	fructose- -bisphosphatase	-0.46785748	0.03491406
BG128321			nitrate transporter	-0.46671236	0.02897103
BI928071			root phototropism protein 3-like	-0.46600047	0.03880424
XM_004239176	TC237061		histidine kinase 3	-0.46501954	0.04135763
AK319766	TC237172		microtubule associated protein type 2	-0.46500635	0.02597909
AK320834	TC236491		lupeol synthase	-0.46371268	0.02073976
AI772296	TC230256		uncharacterized protein	-0.46330457	0.03151194
AW035600	TC229506		e3 ubiquitin protein ligase rie1-like	-0.46250989	0.02533161
XM_004247306			frigida-like protein	-0.46226998	0.031254
AJ785331	TC236561		uncharacterized protein	-0.46201234	0.03995041
AW018272			uncharacterized protein	-0.46135207	0.0218833
BP890621	TC239804		sterol regulatory element-binding protein site 2	-0.46125197	0.03151194
AK327821	TC236361		uncharacterized protein	-0.46085289	0.04442187
AK322785		Solyc10g078250.1.1	70 kda peptidyl-prolyl isomerase-like	-0.45969732	0.031254
AK325708		Solyc12g013820.1.1	ubiquitin conjugating	-0.45962146	0.0479172
BG628024			mads box	-0.4593363	0.0352324
XM_004235486			kda class iii heat shock protein	-0.45808885	0.02521258
AK321277	TC237348		uncharacterized protein	-0.45746716	0.02545931
AK320607			TCP transcription factor 10	-0.45634537	0.0261954
AK321738			uncharacterized protein	-0.45603168	0.031254
AK319572	TC236705		light harvesting chlorophyll a b-binding protein	-0.45442207	0.03290719
AK247762	TC239151	Solyc01g107410.2.1	homeobox protein	-0.45440221	0.03510861
BP906232			chlorophyll a b binding protein	-0.45282303	0.02668256
AB211523	TC226321	Solyc08g075370.2.1	uncharacterized protein	-0.45241737	0.03968076

BI206370			inorganic phosphate	-0.45130768	0.02892259
AK324212	TC227217	Solyc05g026490.2.1	phosphoglucomutase phosphomannomutase family protein	-0.45115439	0.02736339
AK247873	TC219487		uncharacterized protein	-0.45078021	0.04014828
AK247727	TC221999		uncharacterized protein	-0.45069758	0.04830283
BI210411			uncharacterized protein	-0.45022326	0.03968076
FS192950			uncharacterized protein	-0.44998719	0.04473519
AK327731	TC237134	Solyc09g082130.2.1	uncharacterized protein	-0.44940488	0.02409877
GO374183	TC222405	Solyc09g082780.2.1	uncharacterized protein	-0.44924285	0.0340683
BI927471			1-phosphatidylinositol phosphodiesterase-related protein	-0.44912911	0.02338954
AC244940	TC224050		atpase subunit 1	-0.44896024	0.04645831
XM_004234318		Solyc03g026120.2.1	probable methyltransferase pmt15-like	-0.44851226	0.0376923
BF097890			uncharacterized protein	-0.44759447	0.022386
BM412815			dna mismatch repair protein muts	-0.4465273	0.04661723
BI423119			cytochrome p450 like protein	-0.4460411	0.03958396
GO373180	TC236299	Solyc01g073680.2.1	protease inhibitor seed storage lipid transfer protein family protein	-0.44582252	0.0340683
BG628285			e3 ubiquitin-protein ligase sinat5	-0.44510836	0.03911017
NM_001279015		Solyc02g070390.2.1	plastid transcriptionally active 12	-0.44494538	0.03273039
AK319761	TC235921		short-chain dehydrogenase reductase family protein	-0.44461809	0.0378867
XM_004238811			abc transporter g family member 7	-0.44443938	0.0250884
BW687239	TC241571		uncharacterized protein	-0.44428009	0.03172394
AK327428	TC232738		uncharacterized protein	-0.44253749	0.03553866
AK320413	TC229082		uncharacterized protein	-0.44194799	0.03657075
FS191440			atp binding	-0.44191128	0.03210871
AK224678	TC231354		hydrophobic protein lti6a	-0.44187525	0.03765038
AK328815	TC241598		early-responsive to dehydration	-0.44185391	0.02871289
AK321419	TC218178	Solyc00g136260.1.1	e3 ubiquitin-protein ligase atl23-like	-0.441303	0.03595597
BI927119			uncharacterized protein	-0.43992708	0.03423685
BI206991			uncharacterized protein	-0.43941905	0.02715404
AF123258	NP000065		heat-induced chilling tolreance protein HCT6	-0.43928542	0.02540338
XM_004232987	TC244740		trehalose-6-phosphate synthase	-0.43923795	0.03503395
BI209696			class i chitinase	-0.43869877	0.03886765
BT014222	TC237658		pyruvate orthophosphate dikinase regulatory protein	-0.43852502	0.04200443
XM_004245887	TC228474		phosphoribulokinase precursor	-0.43828333	0.0391843
XM_004235250	TC240274		atp binding	-0.43812161	0.03769225
XM_004249865	TC238533	Solyc11g006060.1.1	uncharacterized protein	-0.43804212	0.02787856
AW033803			pathogenesis-related protein 10	-0.43763446	0.03927478
BT013143	TC228153		atp synthase cf1 alpha subunit	-0.43608537	0.04830703
AW034904	TC239912	Solyc12g014150.1.1	uncharacterized protein	-0.4359431	0.0472788
DQ340254	TC217165		auxin response factor 3	-0.43586253	0.03657075
BT013246	TC240076		elongation factor-1 alpha	-0.43493662	0.03204623
AK320203	TC243241		uncharacterized protein	-0.43296025	0.03760724
BT013695			arginine serine-rich splicing	-0.43246425	0.04014828
XM_004233047		Solyc02g071010.1.1	light harvesting chlorophyll a b-binding protein	-0.43166183	0.03345707
BI931815			dna binding	-0.43132367	0.04232291
AK320892	TC218446		uncharacterized protein	-0.43099541	0.03989641
BM408913	TC237493		protein argonaute 4	-0.43075929	0.04070823
AK325149	TC234962	Solyc09g075390.2.1	uncharacterized protein	-0.43032828	0.02627242
AK324619	TC218869		heavy metal transport detoxification domain-containing protein	-0.43013914	0.0488478
AK330003	TC231697	Solyc11g012590.1.1	uncharacterized protein	-0.42995154	0.03042268

AK321930	TC233612		chlorophyll a b-binding protein	-0.42904446	0.0376923
AK326403	TC242653		plastid transcriptionally active 12	-0.42815348	0.04451332
XM_004240345	TC242099		atp-dependent zinc metalloprotease ftsh chloroplastic-like	-0.42797267	0.04470643
GO375405			uncharacterized protein	-0.42772782	0.03215241
BP891849			uncharacterized protein	-0.42714985	0.02897103
BG130624			serine threonine-protein phosphatase bsl2	-0.42552507	0.02621399
AI773206			light harvesting chlorophyll a b-binding protein	-0.42532209	0.04629541
AW617458			serine carboxypeptidase-like protein	-0.42436453	0.0382606
BG130134	TC237159		uncharacterized protein	-0.423784	0.04709
XM_004234193	TC234626	Solyc03g005780.1.1	light harvesting chlorophyll a b-binding protein	-0.42375687	0.03718096
AK319705		Solyc09g090090.1.1	phosphoenolpyruvate carboxylase kinase	-0.42255878	0.04288534
AF328786	TC222118		protein ethylene insensitive 3-like	-0.42192661	0.04830703
AK321736	TC245156	Solyc03g112760.2.1	uncharacterized protein	-0.42157613	0.03522503
XM_004234925	TC239905		uncharacterized protein	-0.42154705	0.0340683
XM_004238409	TC238116	Solyc04g082740.2.1	heat shock protein-like protein	-0.42001466	0.03657075
BT012911			ring-h2 finger protein atl7-like	-0.41940483	0.04399253
AM050814	TC239505	Solyc01g028810.2.1	tcp-1 cpn60 chaperonin family protein	-0.41868354	0.02736339
BP908913		Solyc09g065160.2.1	fzo-like protein	-0.4185662	0.04992153
XM_004249411			dna glycosylase	-0.41742541	0.04245491
AW650716		Solyc03g118630.2.1	uncharacterized protein	-0.41725405	0.031254
AI896197		Solyc11g069990.1.1	disease resistance protein r3	-0.41656994	0.04531512
AK319463	TC227724		phosphate triose-phosphate translocator precursor	-0.41615992	0.04645831
FS191030			homeodomain leucine zipper protein	-0.41596677	0.0382954
GT167532			uncharacterized protein	-0.41592774	0.04714066
CD003544	TC217553		hydroxyproline-rich glycoprotein family protein	-0.41581256	0.04272739
XM_004233698	TC232324		f-box family protein	-0.41556722	0.031254
BG131937			uncharacterized protein	-0.41554429	0.04846895
BP895733			translationally controlled tumor protein	-0.41516191	0.04256384
AK319473	TC219455		ribosomal protein s1	-0.41478962	0.03549013
AW093555			sulfate transporter	-0.41365425	0.0382606
AK319307	TC236916		uncharacterized protein	-0.41354593	0.0320517
AB359913	TC231470	Solyc03g098240.2.1	glutamate decarboxylase	-0.41222109	0.030454
AK325085	TC240978	Solyc08g062960.2.1	heat stress transcription factor a-2	-0.41216608	0.02787856
BI421163			uncharacterized protein	-0.40951484	0.04522901
AF347613	TC217189		sulfate transporter	-0.40926666	0.04844452
BF113448			uncharacterized protein	-0.40878115	0.04792261
BG127006			disease resistance protein	-0.4087583	0.04844452
AJ785173			glycerol-3-phosphate dehydrogenase	-0.4076574	0.03561419
AK324791	TC228696		zeaxanthin epoxidase	-0.40698549	0.03859621
BG129795	TC237419		two-component response regulator arr11	-0.4068722	0.04715727
BF097763			auxin response factor 8	-0.40658858	0.03880424
AK319378	TC235350	Solyc09g065300.2.1	uncharacterized protein	-0.40645651	0.0320517
CD002105	TC231816		uncharacterized protein	-0.40620179	0.03446809
AF154420	TC236199		beta galactosidase 9	-0.4058883	0.03510861
AK326004	TC242548		transcription factor apetala2	-0.4049301	0.04714066
AI896069			uncharacterized protein	-0.40451997	0.03814159
BE354262	TC240213		udp-glucose pyrophosphorylase	-0.40451867	0.03962397
BI930115	TC223942		short-chain dehydrogenase reductase	-0.4032678	0.03914168
AK322281			somatic embryogenesis receptor kinase	-0.40298293	0.04897428

BP887076			uncharacterized protein	-0.40295065	0.04406544
FS181976			uncharacterized protein	-0.40220939	0.0340683
AI898968			uncharacterized protein	-0.40179897	0.0488478
AB217916	TC217195		atp-dependent zinc metalloprotease ftsh chloroplastic-like	-0.40153466	0.04830117
XM_004230269	TC240432	Solyc01g100650.2.1	haloacid dehalogenase-like hydrolase domain-containing protein	-0.40144758	0.04709191
BG134006	TC236508	Solyc07g064240.2.1	early nodulin-like protein	-0.4006306	0.02966005
XM_004230865	TC239798		btb poz domain-containing protein at1g63850-like	-0.40048461	0.031254
AK328294	TC225616	Solyc03g033470.2.1	s-adenosyl-l-methionine-dependent methyltransferase domain	-0.39953221	0.0434884
EU021291	TC217143		phototropin 2	-0.39924613	0.04123466
XM_004244139		Solyc07g063600.2.1	light-harvesting complex ii protein lhcb3	-0.39831134	0.03657075
BG643643			rna recognition motif-containing protein	-0.39796901	0.04360618
BP893974			uncharacterized protein	-0.39795055	0.03462098
AK329678			scp1-like small phosphatase 4b	-0.39622734	0.04758507
AK319457	TC239187	Solyc09g090980.2.1	pathogenesis-related protein 10	-0.39602565	0.04349858
BI206344			uncharacterized protein	-0.39577482	0.04467306
AK323753	TC217272	Solyc07g007120.2.1	homeobox protein knotted-1-like 3-like	-0.39524228	0.02897103
AW220421	TC235729		uncharacterized protein	-0.39348575	0.04657067
ES894678	TC240089		uncharacterized protein	-0.39325865	0.03820904
AW216853			uncharacterized protein	-0.39289672	0.04216354
AK322751	TC224049	Solyc03g096850.2.1	rubredoxin family protein	-0.39283767	0.03880424
BP908405	TC245084	Solyc09g091280.2.1	retinoblastoma-related protein 1	-0.39163123	0.03914168
BM409920	TC233283		uncharacterized protein	-0.39121594	0.0496776
AK328345	TC225010	Solyc06g084610.2.1	uncharacterized protein	-0.39068405	0.03632042
DV103721		Solyc01g005210.2.1	uncharacterized protein	-0.39036823	0.04245151
BP895153			pumilio-family rna-binding domain-containing protein	-0.39026183	0.04117608
AK328263	TC240545	Solyc06g073920.2.1	axial regulator yabby 2	-0.3899815	0.03968076
AK319582	TC244442		33kda precursor protein of oxygen-evolving complex	-0.38968872	0.031254
AK246970	TC229232		uncharacterized protein	-0.38894408	0.03765038
AK328745	TC222228		60s ribosomal protein	-0.38874019	0.0488478
AK319439	TC240183		senescence-associated protein-related	-0.38783547	0.03859621
BI422970	TC232414		histidine decarboxylase	-0.38750149	0.03491406
BG628760	TC245192		nadph-dependent codeinone reductase-like protein	-0.38728431	0.0402595
BI204027	TC239835		sigma factor sigb regulation protein rsbq	-0.38682971	0.04531512
BI930949	TC241421		uncharacterized protein	-0.38632609	0.0352324
AK327177		Solyc06g082320.2.1	uncharacterized protein	-0.38620016	0.03792816
AK320462	TC228683	Solyc12g099200.1.1	invertase inhibitor	-0.38552757	0.03765038
BI928621			chlorophyll a-b binding protein 4 precursor homolog	-0.38544902	0.04456631
XM_004250482	TC239035	Solyc11g011150.1.1	xeroderma pigmentosum group c-complementing protein	-0.38536229	0.03911328
CN641290			serine threonine-protein kinase-like protein ccr3-like	-0.38500889	0.04661723
AK324092	TC226189	Solyc03g117490.2.1	glycerol kinase	-0.38346084	0.03513792
AK324500	TC235459	Solyc08g005560.2.1	binding protein	-0.38316153	0.04017979
AK319524	TC234432	Solyc03g115900.2.1	chlorophyll a-b binding protein 4 precursor homolog	-0.3829534	0.04915587
AM261867	TC223056	Solyc01g098190.2.1	na+ h+ antiporter	-0.38286305	0.0336655
AI896924			beta- -galactosyltransferase sqv-	-0.38249777	0.03510861
BG129762			uncharacterized protein	-0.38149186	0.04336363
AW039683			uncharacterized protein	-0.38141013	0.0382606
BI207577			retrotransposon ty3-gypsy subclass	-0.38137528	0.03880424
BP898990	TC233544		uncharacterized protein	-0.38136323	0.0402219
AK328366			plastidic glucose transporter 3	-0.38062703	0.04014828

AK323916	TC226471	Solyc03g026210.2.1	dihydrodipicolinate reductase	-0.37982891	0.04399253
AK323427	TC243508		alpha tubulin 1	-0.37921431	0.03592504
AW933656			uncharacterized protein	-0.37898215	0.04945286
BG124383			16kda membrane protein	-0.37876605	0.04419626
BG626855			uncharacterized protein	-0.37835569	0.03880424
AW038953	TC241878		uncharacterized protein	-0.37823393	0.04312736
XM_004237772	TC220387	Solyc04g071800.2.1	flavonoid 3-	-0.37779974	0.04601091
AK322637	TC241561		cysteine desulfurase	-0.37747996	0.03914168
AW930124		Solyc07g017590.1.1	uncharacterized protein	-0.37704337	0.04836019
AK319386	TC219667	Solyc03g063240.2.1	root border cell-specific protein	-0.37676559	0.03820845
BG627026			protein acclimation of photosynthesis to environment	-0.37674662	0.03733858
AK320049	TC235633	Solyc03g005000.2.1	uncharacterized protein	-0.37643972	0.04200443
GO372806	TC220807		aspartic proteinase nepenthesin-1	-0.37627801	0.04070823
AK319592	TC223560		light-harvesting complex ii protein lhcb6	-0.37580588	0.03968076
BP906338			ribulose bisphosphate carboxylase	-0.37519236	0.04802333
AK327854			Ubiquitin-activating enzyme E1 domain-containing protein	-0.37403642	0.04830283
GO374202	TC223042		4-hydroxyphenylpyruvate dioxygenase	-0.37389507	0.04919222
BI931968			chlorophyll a-b binding protein 4 precursor homolog	-0.3737146	0.03334823
AW648263			chlorophyll a-b binding protein 4 precursor homolog	-0.3733288	0.03769225
BT014577	TC218324		delta-1-pyrroline-5-carboxylate dehydrogenase 1 protein	-0.37211595	0.03880424
BI921917	TC244507		tubby protein	-0.37165526	0.03749231
BP893679			uncharacterized protein	-0.37165481	0.04353835
AK329490	TC226219		methyladenine glycosylase family protein	-0.37154972	0.0352324
AK327227			uncharacterized protein	-0.37111966	0.04742101
AK247952	TC239993		retroelement pol polyprotein	-0.37092485	0.04725003
XM_004234340		Solyc03g025880.2.1	cleavage and polyadenylation specificity factor 5	-0.37090677	0.04179186
DB682391	TC224489	Solyc07g007020.2.1	wall-associated receptor kinase 2-like	-0.37085625	0.0402595
AK325402	TC237801		heat stress transcription factor a-1	-0.36881892	0.04542311
AK321969	TC244131	Solyc07g043160.1.1	lignan glucosyltransferase	-0.36388098	0.04522901
AK320475			gtp cyclohydrolase ii	-0.36353683	0.0394046
AK326247	TC224781	Solyc10g085810.1.1	uncharacterized protein	-0.36345083	0.03893604
AW617395			uncharacterized protein	-0.36269424	0.04530457
AW036157			thioredoxin h2	-0.3625621	0.03418529
AK326627	TC231580		RHF2A1; protein binding / zinc ion binding	-0.36219392	0.04470643
BF098222			uncharacterized protein	-0.36201487	0.03968271
BG128633			33kda precursor protein of oxygen-evolving complex	-0.36191071	0.03780872
BT013631			protein iq-domain 31-like	-0.36158081	0.03859621
AK321267	TC240838		blue copper protein	-0.36072063	0.04704155
AI775975			stem-specific protein	-0.36047303	0.04017979
BI421716	TC234011	Solyc12g099700.1.1	Lipoyl synthase	-0.36015105	0.04968022
GT168598	TC242000	Solyc06g082940.2.1	photosystem i reaction center subunit xi	-0.35936505	0.0479172
AK325633	TC220517	Solyc05g008920.2.1	uncharacterized protein	-0.35934	0.04017979
AK328037	TC232787		trx fold-containing protein	-0.35932182	0.04496274
CD002305	TC243233		uncharacterized protein	-0.35874777	0.04135763
XM_004240123	TC217646		4-hydroxyphenylpyruvate dioxygenase	-0.35854252	0.0433385
BI935751	TC225009		gag and pol identical	-0.35835858	0.04844452
DB701784	TC245234		uncharacterized protein	-0.3582213	0.04683372
BW687846	TC231814	Solyc11g072540.1.1	transducin wd40 domain-containing protein	-0.35734276	0.03914168
AK321201	TC226908	Solyc06g083420.1.1	uncharacterized protein	-0.35648635	0.04864346

BI928326			uncharacterized protein	-0.35315864	0.04897428
BM411005			uncharacterized protein	-0.35303157	0.04624782
AW223023			eukaryotic translation initiation factor 5b	-0.35250606	0.04714066
AK247769			uncharacterized protein	-0.35248599	0.04830703
BP897648			light-harvesting complex i chlorophyll a b binding protein 1	-0.35171627	0.04288534
AW932557	TC235636	Solyc03g095310.2.1	cytochrome p450	-0.35062425	0.04830283
DB708884	TC232014	Solyc06g065490.2.1	domain-containing protein	-0.3499393	0.04830283
CK715512			histone-lysine n- h3 lysine-9 specific suvh9	-0.34968244	0.03880424
BE450133			uncharacterized protein	-0.34935579	0.04802333
DB679602	TC239043	Solyc10g047130.1.1	rna recognition motif family expressed	-0.34913342	0.04968022
AK246319			thylakoid lumen kda protein	-0.34887865	0.04297324
AK320448	TC221090	Solyc03g121920.2.1	solute carrier family 25 member 44-like	-0.3487462	0.03880424
AK320831	TC238528		hxxxd-type acyl-transferase-like protein	-0.34741745	0.03886765
U83708	TC241686		farnesyltransferase beta subunit	-0.34736916	0.04530457
BI929455	TC235551		uncharacterized protein	-0.34723606	0.04897428
BI207919			lim domain-containing protein	-0.34568888	0.0391856
AK320157			translation initiation factor if-3	-0.34559312	0.04017979
DB684193	TC236660		thioredoxin m4	-0.34384387	0.04092675
AK322836			gtp-binding protein	-0.34270362	0.04344963
DB720036	TC241530		uncharacterized protein	-0.3404077	0.04531512
AW037578		Solyc06g034120.2.1	quinone oxidoreductase	-0.33914509	0.04046689
AI484567			uncharacterized protein	-0.33793004	0.04929429
AW616828			uncharacterized protein	-0.33771389	0.04650693
XM_004234432	TC221437		at4g04770 t4b21_16	-0.33623244	0.04130896
AJ831881			23s ribosomal rna	-0.33581857	0.04802605
FS200032			glutamate binding protein	-0.33371561	0.04915587
AK323146	TC218899		uncharacterized protein	-0.3323939	0.04542311
BM413605			Nuclear transcription factor Y subunit A-3	-0.3323296	0.04213426
BP881200			uncharacterized protein	-0.33177889	0.0433385
DB717269			atp synthase subunit	-0.32774916	0.04798909
XM_004235672		Solyc03g120000.2.1	RING finger protein	-0.32380254	0.04897428
BI422737	TC242220		uncharacterized protein	-0.32268687	0.04424732
AK247192	TC230419		cyclin-dependent kinase f-4-like	-0.31723751	0.04728321
AJ784533			uncharacterized protein	-0.31182591	0.04984299

Supplementary Table S3. Primer sequences used for Anchor-PCR, Genotyping, and qRT-PCR analysis

A. Primers used for Anchor-PCR analysis

	Primer name	Primer sequence (5’-3’)
	Adaptor-1	CTAATACGACTCACTATAGGC
	Adaptor-2	CTATAGGGCTCGAGCGGC
	Adaptor-3	AGCGGCGGGGAGGT
	Anchor-Right-1	CAGACGCCTTGGTTAGGTTG
	Anchor-Right-2	GTTCCCCTTCTGGAAGATCG
	Anchor-Right-3	GGGTGTTCTGTCGTCTCGTT
	Anchor-Left-1	CAAAGGCAACCAAACAAACA
	Anchor-Left-2	GCGTAGTGACCCGACAAATAA
	Anchor-Left-3	AATTATGCTCAAACATTTCCATGA

B. Primers used for Genotyping analysis

	Primer name	Primer sequence (5’-3’)
	Genotyping-F	TGGCCAAAACACTTCAATTTC
	Genotyping-R	TCAACCAATCAAAACTCGACA
	T-DNA-R	GGCAACCAAACAAACAATGA

C. Primers used for qRT-PCR analysis

Gene locus identity[a]	Primer name	Primer sequence (5’-3’)
Solyc01g056940.2.1	S01g056940-F	CCAAGATCCAGGACAAGGAA
	S01g056940-R	AAATCAAACGCTGCTGGTCT
Solyc02g078150.2.1	S02g078150-F	TGCCGCAACTGTCTTATGAA
	S02g078150-R	CCGGTGAGCTTTTCCATTTA
Solyc02g081670.1.1	S02g081670-F	TACCTCCTTGTCCATTTTTG
	S02g081670-R	AACTCTCCCCCTCCAACTC
Solyc03g119370.1.1	S03g119370-F	ACCTGGAAGAACGGACAATG
	S03g119370-R	GAGGCGTTGATGACTGTGAA
Solyc05g012020.2.1	S05g012020-F	AACATCATGGCATTGTGGTG
	S05g012020-R	TGTTGATGGTGCTGCATTTT
Solyc05g015750.2.1	S05g015750-F	TTCATTGCGATATGGTAACCT
	S05g015750-R	CATCCAACGTGAACTTAGACGA
Solyc05g056620.1.1	S05g056620-F	TCTCTGAACTGCAGAAAAAGGA
	S05g056620-R	TAGTTTGCTGGTGCCATTCA
Solyc06g007180.2.1	S06g007180-F	GAGCCTGACAGTTCCTGGAG
	S06g007180-R	TAGCAGCCCTACCAGAAGGA
Solyc06g007580.1.1	S06g007580-F	ACACCGTTTCATGCAAAACA
	S06g007580-R	GCCTGATTCGATGGAGGTAA
Solyc06g068500.2.1	S06g068500-F	ACGGCTATGGGAATGATGAG
	S06g068500-R	GTCTTTTTGTTTGCGCGATT
Solyc06g082030.2.1	S06g082030-F	CAAACGCACAGCTCCAATAA
	S06g082030-R	GCATGTTCTTCAATTTGGTTCA
Solyc08g006740.2.1	S08g006740-F	TCTTCAGCAAGCAGGAATCA
	S08g006740-R	ATAATTGCCAACGACGAACG
Solyc10g076740.1.1	S10g076740-F	AACAGCAGAAGCAAGCAAAG
	S10g076740-R	CCAAGTCCAAATCAGCAACA
Solyc12g006380.1.1	S12g006380-F	TCTCAAAGAAATGGGATGTGC
	S12g006380-R	TTGCAATTTCTGGTTGTGGA
Solyc12g036390.1.1	S12g036390-F	CATGCAAAAACTTGTGAGATGG
	S12g036390-R	GAAGGCATTCCTCACCAAAT
Solyc12g098590.1.1	S12g098590-F	TGGACCGATCAAATGACAAA
	S12g098590-R	CCTTTTACCCCTCCATCCAT

[a] Gene locus identity based on ITAG release 2.4 official annotations on the SL2.50 genome build by the International Tomato Annotation Group (ITAG). The SOL Genomics Network (SGN) http://solgenomics.net/
